# Single-shot 3D imaging with point cloud projection based on metadevice

**DOI:** 10.1038/s41467-022-35483-z

**Published:** 2022-12-21

**Authors:** Xiaoli Jing, Ruizhe Zhao, Xin Li, Qiang Jiang, Chengzhi Li, Guangzhou Geng, Junjie Li, Yongtian Wang, Lingling Huang

**Affiliations:** 1 Beijing Engineering Research Center of Mixed Reality and Advanced Display, School of Optics and Photonics, Beijing Institute of Technology, Beijing, 10081 China; 2 Beijing National Laboratory for Condensed Matter Physics, Institute of Physics, Chinese Academy of Sciences, Beijing, 100191 China

**Keywords:** Metamaterials, Micro-optics, Imaging and sensing

## Abstract

Three-dimensional (3D) imaging is a crucial information acquisition technology for light detection, autonomous vehicles, gesture recognition, machine vision, and other applications. Metasurface, as a subwavelength scale two-dimensional array, offers flexible control of optical wavefront owing to abundant design freedom. Metasurfaces are promising for use as optical devices because they have large field of view and powerful functionality. In this study, we propose a flat optical device based on a single-layer metasurface to project a coded point cloud in the Fourier space and explore a sophisticated matching algorithm to achieve 3D reconstruction, offering a complete technical roadmap for single-shot detection. We experimentally demonstrate that the depth accuracy of our system is smaller than 0.24 mm at a measurement distance of 300 mm, indicating the feasibility of the submillimetre measurement platform. Our method can pave the way for practical applications such as surface shape detection, gesture recognition, and personal authentication.

## Abstract (web summary)

The authors present a single-shot 3D imaging approach utilizing carefully designed point clouds projection based on a metasurface device. They show submillimeter depth accuracy and demonstrate the potential for hand gesture detection.

## Introduction

Three-dimensional (3D) imaging can perceive real-world 3D objects and reconstruct detailed features of spatial information. Due to the digital description capability of 3D imaging^1,2^, it plays a key role in numerous applications, including artificial intelligence, virtual reality, robot navigation, heritage conservation, and industrial design and inspection. In recent years, structured light techniques^3,4^ have been developed rapidly in both the scientific and industrial community with excellent performance in surface measurement, fast short- and mid-range distance measurement, and high accuracy. However, the size of traditional projector devices is limited because the refractive lens, and more components impose more difficulties on precise system construction, which results in a technological and manufacturing challenge to achieve compact devices. Diffractive optical elements (DOE) may only generate point clouds within a relatively small field of view owing to the large pixel sizes compared with light wavelengths^5,6^. Meanwhile, a 3D reconstruction algorithm for different platforms needs to be strictly designed in association with the corresponding hardware according to the accuracy, speed, and data capacity requirements. Therefore, both simple devices and corresponding reconstruction algorithms are imperative for single-shot 3D imaging.

Metasurfaces^7^, considered as the 2D equivalents of 3D metamaterials, are artificial optical surfaces that enable flexible modulation of the amplitude^8,9^, phase^10,11^, and polarization^12,13^ of the light field. They therefore provide novel platforms for numerous applications in holography display^10,14–16^, conformal optics^17–19^, and beam shaping^20–22^. Their features of miniature size, large numerical aperture, full space control, and multifunctional capability^23,24^ have accelerated their applications in 3D imaging^10^. In particular, both metalens array^25^ and bifocus metalens^26^ have been utilised in passive 3D positioning and imaging techniques, showing great potential for millimetre-scale, low-power platforms. Nevertheless, there are several difficulties in the above technique based on imaging metalens^27–29^, including limited field of view (FOV), depth of field, and image resolution. Some devices based on metasurfaces related to active 3D imaging techniques have been proposed, and they all have a relatively large FOV compared with DOE benefitting from the subwavelength size. A periodic metasurface for generating point clouds in large angular space has been demonstrated^30^ by optimizing the intensity uniformity of selected diffraction orders, indicating its potential spatial coding capability with the advantage of polarisation multiplexing. Meanwhile, a Dammann grating based on metasurface has been demonstrated to replace DOEs with a larger FOV^31^, but it only offers limited diffraction orders to expand the collimated laser pattern from the vertical-cavity surface-emitting laser (VCSEL) array. Hence, integrating metasurfaces with laser sources can greatly enhance compactness and scalability^32,33^, paving the way for the design of versatile on-chip optoelectronic devices.

**Fig. 1:**
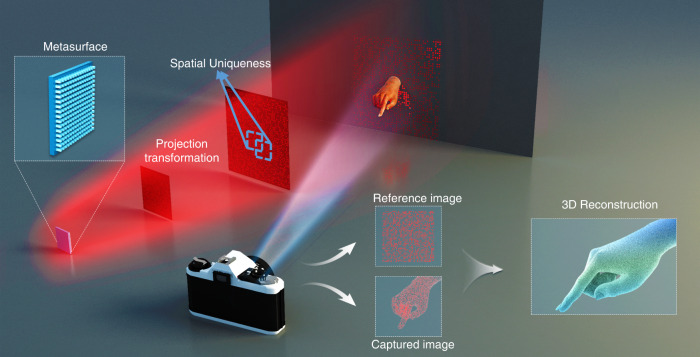
Schematic of single-shot 3D imaging based on metasurface projection. Compact projector based on single-layer metasurface is used to project the designed point cloud pattern, and the reflected images are captured by single-shot camera. The projection pattern in the Fourier domain satisfies the projection transformation, and the in-plane uniqueness is designed by engineering the metasurface. The matching of the point cloud pattern in the capture image and the reference image is used to reveal depth information based on triangulation.Consequently, depth estimation is achieved by taking advantage of in-plane and out-plane spatial uniqueness, which can be applied in gesture recognition.

In this paper, we introduce a metasurface to project the designed pattern and present a complete computational architecture to obtain 3D information (Fig. 1), which constitute an effective 3D imaging approach. The metasurface is used to project the judiciously designed pattern in Fourier space, and a 3D reconstruction operation is proposed based on the triangulation principle^34^. The FOV design and number of projection points are also discussed for high-performance imaging. We also propose and analyse a complete computational framework that effectively achieves an accurate single-shot 3D reconstruction. Consequently, the depth accuracy and 3D reconstruction of different scenes using the proposed method are experimentally demonstrated. Such method is a promising approach for the future of flat optical devices in the consumer electronics and industrial vision markets; the method reduces the alignment complexity, vibration sensitivity, and manufacturing complexity of current optical imaging solutions.

## Results

### Metasurface-based projection for single-shot 3D imaging

It is well known that by engineering the material, geometry, and inner resonance effect of individual nanostructures, one can control the phase, amplitude, and polarisation of the transmitted wavefront at subwavelength scales, allowing the metasurface to become a functional device in either the real space or frequency domains. Because Fourier holograms based on metasurfaces have large numerical apertures owing to subwavelength scales, the projector based on a single metasurface has a small throw ratio and long projection range. Fig 1 depicts the mechanism of 3D imaging using a compact projector based on a single-layer metasurface. The projection pattern is composed of random point clouds, and the local pattern in a rectangular window (blue window in Fig. 1) is unique in the entire projection plane, which can be identified from the spatial distribution. Meanwhile, the projection pattern is clear and satisfies the projective transformation in the entire Fraunhofer diffraction region (see Supplementary Note 1), offering a complete and accurate mathematical description of the structured pattern in a 3D space. Therefore, by combining different positions of the metasurface and the camera, it is possible to measure the 3D shape of an object based on the principle of triangulation.

It must be noted that the illumination pattern generated by the metasurface may not be identical to the design pattern owing to speckle noise. To overcome this challenge, a calibration and reconstruction operation based on the reference plane and auxiliary planes is proposed (see Supplementary Notes 3 and 4). A reference plane and two auxiliary planes are required to record the practical pattern and establish the relationship between depth and pattern shift based on cross-ratio, which is one of the most important invariants in the perspective transform. Depth information was obtained by combining the pattern offset or deformation of the captured images; depth information is obtained as shown in Fig. 1. The search operation of the corresponding pattern between the target and reference images is critical for depth calculation, and a corresponding matching algorithm is proposed based on pattern characteristics. Therefore, our method can achieve single-shot 3D imaging, which is very useful for human-computer interaction, such as gesture recognition, as shown in Fig. 1.

### Metasurface design and characterisation

**Fig. 2:**
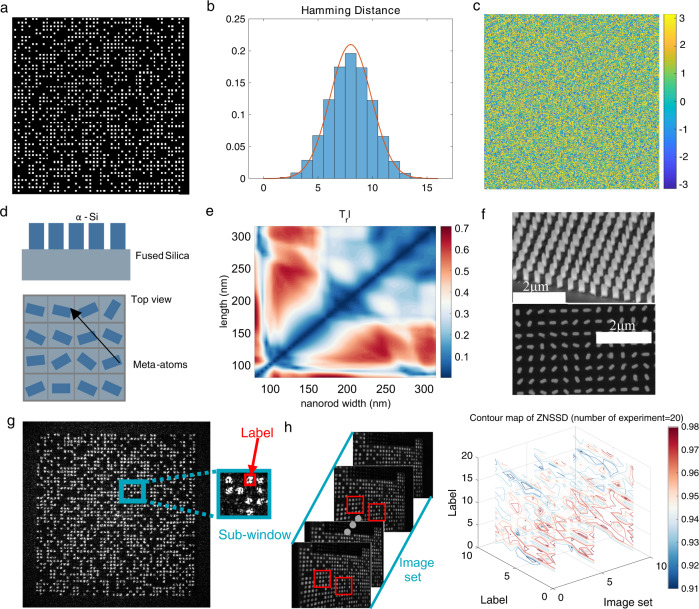
Design, manufacture, and detection of metasurface. **a** Design of projection pattern. The pattern is a type of pseudo random code. **b** Hamming distance distribution. This can be regarded as an approximate Gaussian distribution, which is similar to the orange curve generated with Gaussian expression. **c** Phase profile calculated using GS algorithm. **d** Nanopillars and their spatial distributions based on geometric phase principle.**e** Transmission coefficients obtained by sweeping the geometric parameters of a nanopillar within a unit cell.**f** Top-view and side-view SEM images. **g** Holographic reconstructed image. For the convenience of similarity calculation, we define the sub-window and label, which are shown in the enlarged view. **h** Correlation of the image set under different depths. Ten images are randomly captured at different depths, and the number of times that the measurements were repeated is 20. Three contour maps of ZNSSD with different depths are displayed.

To build a single-shot 3D imaging mechanism, the uniqueness of the local pattern in the entire projection pattern must be satisfied; thus, there is no need for another pattern to determine the corresponding points. M-array code^35^, as a type of pseudo-random coding, can cause the pattern of any sub-window to appear only once in the whole pattern, achieving local uniqueness of the pattern. Therefore, M-array coding is used to design the projection pattern, as shown in Fig. 2a. The total number of spots is designed to be 1201, which can be further improved by large-area processing. The density of the projection pattern, defined by the ratio of the total area of bright spots to the area of the projection pattern, is 50%; a large information capacity guarantees precise calculation of the depth value. First, the uniqueness of every bright spot is demonstrated by the Hamming distance^36^, which is quantified by,

1 $$\begin{array}{c}H({i}_{1},\,{j}_{1};{i}_{2},\,{j}_{2})=\mathop{\sum }\limits_{i=0}^{n-1}\mathop{\sum }\limits_{j=0}^{n-1}\delta (p({i}_{1}+i,{j}_{1}+j),\, p(({i}_{2}+i,{j}_{2}+j)))\\ \delta (a,b)=\Bigg\{\begin{array}{c}0\quad\,a=b\\ 1\quad\,\,a \, \ne \, b\hfill\end{array}\end{array}$$

where *H*(*i*_1_, *j*_1_; *i*_2_, *j*_2_) is the Hamming distance between two sub-windows, each centred at the points (*i*_1_, *j*_1_) and (*i*_2_, *j*_2_) with the window size *n* × *n*, which can serve as an indicator of the pattern difference between two sub-windows in the projection plane. The maximum Hamming distance is *n* × *n*. A larger Hamming distance *H*(*i*_1_, *j*_1_; *i*_2_, *j*_2_) indicates a larger diversity between the two spots, which can be distinguished robustly under severe noise. For convenience, the statistical histogram of *H*(*i*_1_, *j*_1_; *i*_2_, *j*_2_) is shown in Fig. 2b with *n* = 4, which quantifies the local uniqueness in the entire projection plane. As shown, zero Hamming distance does not exist, and a Hamming distance below 4 has a proportion of less than 0.05, indicating that bright spots can be accurately determined by the space information of the adjacent spots. Therefore, taking advantage of the uniqueness of the local spatial information, a fast-matching algorithm to determine the corresponding spots can be designed easily.

Metasurface holography, benefitting from cutting-edge nanotechnology, has excellent performance, such as high precision of reconstructed images, freedom from undesired diffraction orders, and large space-width products. In particular, because the reconstructed image is located in the far field of the metasurface, Fourier holography has a large depth of field. Phase modulation based on metasurfaces can be easily achieved, and geometric metasurfaces enable superior phase control with the advantages of broadband performance, robustness against fabrication errors, and the helicity switchable property, facilitating the encoding procedure. Meanwhile, the Gerchberg-Saxton (GS) algorithm was used to calculate the phase hologram (see Supplementary Note 2), which was then encoded on the geometric metasurface as a physical implementation. To ease the fabrication challenge, the phase profile was discretised with eight phase levels, as shown in Fig. 2c.

We choose amorphous silicon, which can be obtained using a standard nanofabrication method. As shown in Fig. 2d, amorphous silicon nanopillars with different orientation angles are arranged on a fused silica substrate to achieve the desired phase profile. To cover the phase shifts from 0 to 2π with high efficiency, the period and height of the nanopillars are chosen as 316 and 600 nm, respectively. A rigorous coupled wave analysis (RCWA) method is used to optimise the 2D parameters of the nanopillars at an operating wavelength of 633 nm. The simulated results for the transmission coefficient of the polarisation conversion efficiency are shown in Fig. 2e (See the efficiency analysis in Supplementary Note 2). Then, the length and width are determined as 180 and 80 nm, respectively, to maintain a high transmission efficiency. By engineering planar nanostructures, the desired phase profile can be converted into a diverse orientation distribution. The fabricated metasurface is composed of 1578 × 1578 nanopillars using electron beam lithography and reactive ion etching, and the corresponding scanning electron microscopy images with side and top views are shown in Fig. 2f.

To obtain the properties of the illumination pattern based on the metasurface, we use a conventional optical scheme to capture holographic images (see Methods section). The reconstructed image, shown in Fig. 2g, has a high degree of similarity with the design pattern, but also possesses some speckles. Speckles are primarily generated by fabrication errors and unavoidable coherent laser noise. Nevertheless, such speckles may offer additional information in the inner region of every spot; more details can be obtained in a favourable way. For the completeness of the proof, the zero-normalised sum of squared difference coefficient (ZNSSD)^37^ calculated by 300 different labels with their corresponding labels at three different depths are shown in Fig. 2h, demonstrating the similarity of speckle patterns at different depths. The definition of the ZNSSD is as follows:

2 $${C}_{ZNSSD}=\mathop{\sum }\limits_{x=1}^{M}\mathop{\sum }\limits_{y=1}^{N}{\left[\frac{f(x,y)-{f}_{m}}{\sqrt{\mathop{\sum }\nolimits_{x=1}^{M}\mathop{\sum }\nolimits_{y=1}^{N}{[\,\,f(x,\,y)-{f}_{m}]}^{2}}}-\frac{g(x^{\prime},\,y^{\prime} )-{g}_{m}}{\sqrt{\mathop{\sum }\nolimits_{x=1}^{M}\mathop{\sum }\nolimits_{y=1}^{N}{[\,g(x^{\prime},\,y^{\prime} )-{g}_{m}]}^{2}}}\right]}^{2}$$

where *f*(*x*, *y*) and *g*(*x’*, *y’*) are the grey level intensities at the coordinates (*x*, *y*) and (*x’*, *y’ *), respectively, in the selecting label of two images in different observation planes. *f*_*m*_ and *g*_*m*_ are the mean grey-level intensities in the subset. *M* and *N* are the sizes of the subsets along the *x* and *y* directions, respectively. A few representative ZNSSD contour maps are shown in Fig. 2h, with more images and contour maps shown in Supplementary Note 5. Fig 2h illustrates that the ZNSSD values are all greater than 0.9 at different depths, so the similarity can be used to determine the corresponding pixels in the inner region of the spots.

### Matching algorithm

The proposed matching algorithm consists of a feature-based initial matching algorithm and an area-based fine matching algorithm, leveraging the spatial uniqueness and speckle features of labels, respectively. This method combines both the robust and effective label matching of feature domain transformation, and dense pixel correspondences of geometrical area deformation, efficiently leading to accurate and dense matching results. The matching process can be modelled as the establishment of correspondence relations between the deformed image and the reference image with the constraint of surface continuity, which can be described mathematically as follows:

3 $$\begin{array}{c}\{{{u}_{i}}^{\ast }(x,y)\}={{{\mbox{arg}}}}\,\min \mathop{\sum }\limits_{i=1}^{n}{|f(x,y)-g({u}_{i}(x,y))|}_{2}^{2},\,(x,y)\subset {\varOmega }_{i}\\ st\,{F}_{{{{{{\rm{c}}}}}}}({u}_{i}(x,y)),\,(x,y)\subset {\varOmega }_{i}\end{array}$$

where *u*_i_^*^(*x*, *y*) is the optimal estimation of the correspondence functions for each local correspondence estimation *u*_*i*_(*x, y*) in subregion Ω_*i*_. *n* is the number of subregions, and *f* and *g* are the reference and deformed images, respectively. *F*_c_ is the constraint operator that guarantees the global continuity and compatibility of *u*_*i*_ (*x, y*).

**Fig. 3:**
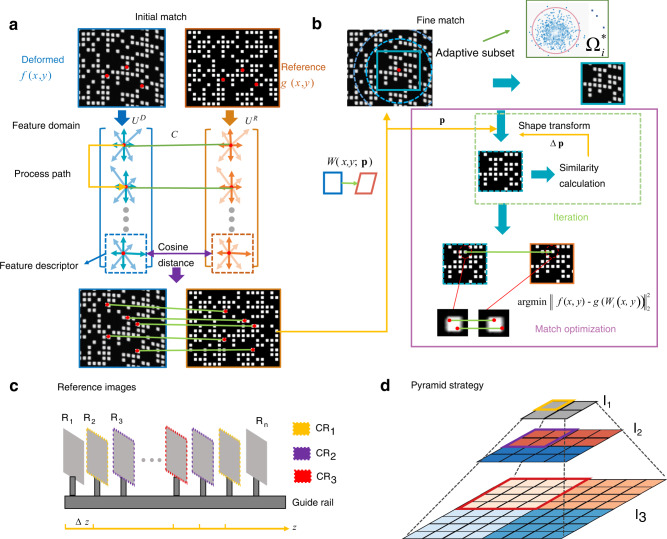
Computational algorithm and strategy of correspondence search based on pattern features. **a-b** Computational architecture of correspondence search algorithm. **a** Initial match, which utilises feature domain transform to perform similarity calculation with a designed path as a constraint of surface continuity, achieving the correspondence search of all labels. *U*D and *U*R are the label sets of the deformed and reference images, respectively, and the cosine distance is used to match them. **b** Fine match. Fine match aims to obtain more sophisticated results with shape function optimisation and region constraint, leveraging the instrinsic features of the labels. The initial results are used to calculate the coarse deformation function *W*. **c-d** Multi-resolution search strategy. **c** Capture of reference images. The reference images are captured along the z-direction with the same intervals; the equality of these inttervals is achieved with a precise guide rail. **d** Pyramid strategy. The high-resolution image named I3 is the original image, and the other two images named I2 and I1 are obtained by wavelet transformation. The candidate reference (CR) in **c** corresponds to the image block in **d** with the same color of the border.

The operation of the initial match relies on the spatial uniqueness of labels validated by the Hamming distance, allowing transformation to the feature parameter space with handcrafted feature descriptors for label matching (the comprehensive theory, implementation, and demonstration of the initial matching algorithm are shown in Supplementary Note 6). Feature descriptors consist of simple vectors for the discriminative representation of each label. Formally, the initial match can be expressed as

4 $$C={M}_{CD}{({U}_{{{{{{\rm{fd}}}}}}}(g),{U}_{{{{{{\rm{fd}}}}}}}(\,f))\big|}_{N=(U,\varGamma )}$$

where *U*_fd_ is the feature descriptor and *C* is the correspondence matrix. *M*_*CD*_ is the match operator based on the cosine distance measurement applied to *U*^*D*^ and *U*^*R*^, which are the label sets of the deformed image and the reference image, respectively, as shown in Fig. 3a. The cosine distance is widely used as a metric of vector similarity. Simultaneously, a set of spatial neighbour labels *N* = (*U*, *Γ*) is constructed as the designed constraint. Each label *U* is associated with the corresponding neighbour label *Γ*. *N* determines the process path of labels in *U*^*D*^ based on geometrical cues that utilise the neighbour information of the processed labels. The labels are precisely matched to the corresponding labels of the reference images in an indirect manner by the initial matching algorithm.

Because the initial match result offers robust label correspondence, the coarse correspondence estimation can be obtained using the labels involved in the local area. Combined with the speckle features of the inner region of the labels, we address the fine match as an optimisation problem:

5 $${{W}_{i}}^{\ast }={{\mbox{arg}}}\,\min {|f(x,y)-g({W}_{i}(x,y;{{{{{\bf{p}}}}}}))|}_{2}^{2},\,(x,y)\subset {\varOmega }_{i}^{\ast }({{{{{\bf{p}}}}}}),\,i=1,2,\cdots n$$

where **p** is the initial deformed parameter calculated by the initial match results *C* (see Supplementary Note 6). *W* (*x*, *y*; **p**) is the shape function relative to the reference image and describes the mathematical relationship between the spatial position of the deformed region and the reference region. Ω_i_* is the *i*th subarea, as shown in Fig. 3b. Equation (5) aims to find the shape function based on minimising the dissimilarity between the deformed and reference images after shape transformation in all adaptive subareas (the match accuracy with subarea size is discussed in Supplementary Note 7). In particular, the operation of the adaptive subarea is conducted by progressively selecting the local region Ω_*i*_ based on the geometry transform with respect to **p**, discarding those outliers which have dissimilar geometry transformations. The match optimisation subsequently yields an elaborate match using the inverse-compositional Gauss–Newton (IC-GN) algorithm^38^ with the initial parameter **p**, which aims to minimise the dissimilarity by the iteration of the shape function increment ∆**p** in the local subset Ω_*i*_. Essentially, the initial deformed parameter **p** plays a significant role in constructing the constraint of spatial continuity, which is used to gradually achieve an appropriate subarea for a stable support domain and to constrain the solution space of the shape function. Finally, the IC-GN algorithm finds an appropriate correspondence solution to satisfy the constraints, achieving a pixel or sub-pixel match with the fine matching algorithm.

### Multiresolution search strategy

A multi-resolution search strategy is proposed, as shown in Fig. 3c–d, to balance the matching accuracy and computational efficiency. Multiple images are captured along the *z*-direction at the same intervals, as shown in Fig. 3c, which can all be regarded as reference images. The multiresolution search method utilises the low-resolution images to obtain a coarse depth map, which can be used backwards to select the most suitable reference images; thus, high-resolution images can be used to calculate a more precise depth with the updated reference images. The operating principle of the multiresolution search method is described in a more condensed notation as follows:

6 $${{{{{{\rm{Z}}}}}}}_{i}	={F}_{{{{{{\rm{Rec}}}}}}}({{{{{{\rm{I}}}}}}}_{i},\,{{{{{{\rm{CR}}}}}}}_{i})\\ {{{{{{\rm{CR}}}}}}}_{i}	=\Bigg\{\begin{array}{c}{{{{{{\rm{CR}}}}}}}_{1},\qquad i=1\hfill\\ \{{{{{{{{\rm{CR}}}}}}}_{i}}^{\ast }\}=\,\min|{{{{{{\rm{Z}}}}}}}_{i-1}-{{{{{{\rm{Z}}}}}}}_{C{R}_{i-1}}|\,i \,\, > \, 1\end{array}$$

where CR is the candidate reference image, I is the deformed image, *F*_Rec_ is the reconstruction operator, and Z is the depth after the reconstruction calculation. First, the deformed image is transformed into multiresolution images named I_1_, I_2_, and I_3_ by wavelet transform, which we call the pyramid strategy, as shown in Fig. 3d. The low-resolution image I_1_ is first used to calculate the coarse depth map Z_1_ by two fixed planes named CR_1_, which have a yellow border in Fig. 3c. Then, the two planes nearest to the coarse depth Z_1_ are chosen as new reference planes CR_2_, which have a purple border in Fig. 3c. The same operation is conducted for image I_2_. Finally, candidate planes move closest to the reference image of real depth, and the depth results with the original resolution image I_3_ are more precise because of the higher similarity with the reference image. Consequently, the pyramid sampling strategy can be used for coarse-fine search to improve the measurement accuracy and reduce the measurement uncertainty at the expense of speed. However, sacrifice is not severe because of the relatively low computational cost of low-resolution images (We also discuss the acceleration method in Supplementary Note 8).

### Depth–accuracy demonstration

To analyse the depth accuracy, a camera with a mounting angle of 30° relative to the baseline is used to capture the images of test objects at a distance of 300 mm from the metasurface. The resolution of the camera is 2448 × 2048 pixels, and the focal length of the imaging lens is 16 mm. Five groups of two different flat objects were captured with our proposed 3D imaging device, and the height differences between the two flat objects were used to achieve the evaluation, compared with the known thickness of 1.69, 2.00, 2.74, 3.69, and 4.00 mm.

**Fig. 4:**
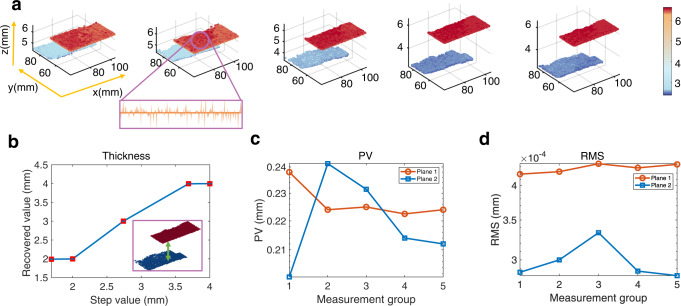
Measurement accuracy validation with different setups. **a** Recovered point cloud images with five different height differences. We have measured five flat objects (ceramic slabs) with different thicknesses and one common flat object, and their thicknesses are 1.69, 2.00, 2.74, 3.69, and 4.00 mm, respectively. The area of the measured scene is ~80 mm 100 mm. The data fluctuation of the point cloud can be used for the evaluation of the reconstructed result, in which a local line data is shown in the enlarged view of the second setup. **b** The recovered thickness. The recovered thickness is calculated using the difference in the average value of the two planes, which is shown as the green arrow in the inset sketch map. The thickness error compared with real values for the five groups are 0.19, 0.01, 0.2, 0.12, and 0.02 mm, respectively. **c-d** The PV and RMS values of the planes of five setups. The legend of plane 1 represents the higher plane in the five setups, and plane 2 represents the lower one. The PV and RMS values are used to evaluate the planeness. The maximum PV and RMS values are 0.24 mm, and 4.4 10-4mm, respectively.

The reconstruction point cloud images of five setups and error analysis are shown in Fig. 4, which are obtained with the proposed matching algorithm and multi-resolution search strategy (the comparison between the multi-resolution search strategy and fixed reference image is shown in Supplementary Note 8). The error data for five measurement groups are 0.19, 0.01, 0.2, 0.12, and 0.02 mm as shown in Fig. 4b. The maximum error is approximately 0.2 mm at a depth of 300 mm, indicating that the recovered height differences of the two objects are in good agreement with those of known experimental setups. The depth accuracy can be attributed to the spatial uniqueness of the illumination pattern, which combines the principle of triangulation and the proposed matching algorithm. These results quantitatively demonstrate the effectiveness of depth perception with our proposed method, which is very promising for applications in the 3D positioning and imaging of millimetre platforms. Meanwhile, data drift appears in the point cloud of the planes owing to unavoidable measurement errors, and the planeness is evaluated by the peak valley (PV) and root mean square (RMS) values of all recovered points, as shown in Fig. 4c–d. The maximum PV value was 0.24 mm, indicating that the error of individual measurement points caused by noise or boundary was less than 0.24 mm. The maximum RMS value was 4.4×10^−4 ^mm, indicating a good performance resulting from the fine matching with the sub-pixel search method. Therefore, dense and accurate point cloud data can be obtained, demonstrating the potential for accurate and robust 3D information acquisition.

### 3D shape reconstruction for a variety of scenes

**Fig. 5:**
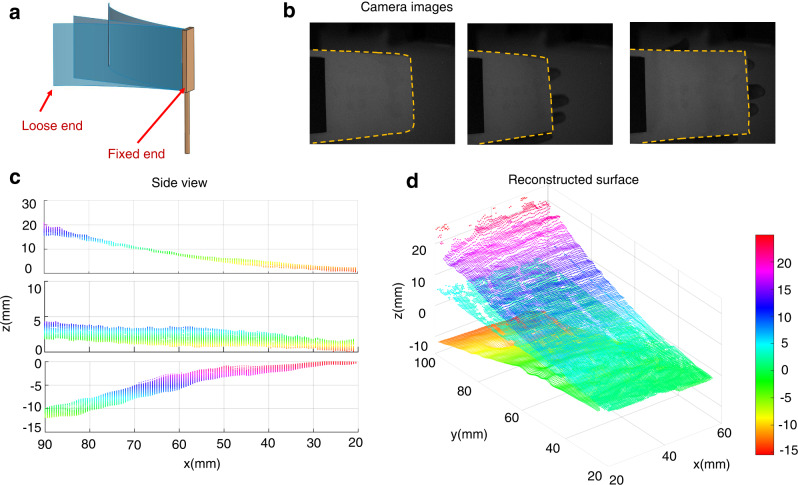
3D imaging of cardboard under three deformation states. **a** Sketch map of deformed cardboard under test. The area of the measured scene is ~40 mm 80 mm. The cardboard is fixed with the splint, and the deformation is excited with the loose end of the cardboard manually. **b** Captured images of cardboard by a camera. The boundary is plotted as the coloured dotted line. **c-d** Side views and 3D geometric maps under three deformation states.

Easily deformable cardboard was used to validate the 3D imaging capability for continuous and low-texture surfaces. Three captured images are shown in Fig. 5b, and the deformation is excited with the loose end of the cardboard by a human hand, as shown in Fig. 5a. The side views (*y*-*z* plane) and reconstructed 3D shapes of the deformed cardboards at three different manual pressures are shown in Fig. 5c, d, respectively. These results verify that the proposed method enables the 3D reconstruction of object vibration and deformation. Meanwhile, the 3D reconstruction ability for low-texture objects renders the technique advantageous for active imaging techniques over passive imaging technique^39^, such as binocular stereo vision and depth from defocus. Note that our method can also work for measured scene with larger object size (Supplementary Note 10).

**Fig. 6:**
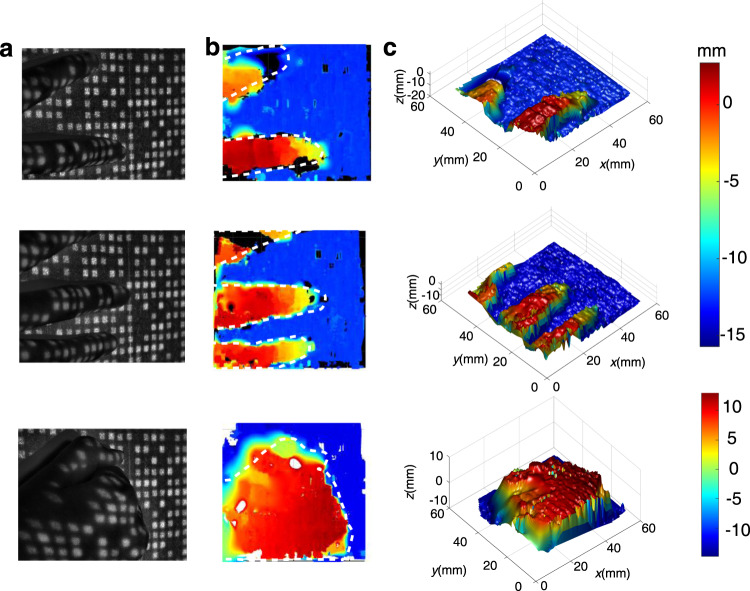
3D imaging for gesture acquisition. **a** Captured images of three gestures. The images have been cropped to shown the gesture. The cropped area of the measured scene is ~60 mm 60 mm. **b** Depth maps. The depth map shows the similarity with the 2D (two-dimensional) contour of captured images, such as the position and orientation of fingertips or hand, and the white curves denote the outline of fingertips and hand of the captured images. **c** Point cloud maps of the reconstructed gestures. The depths of both the fingers and the background are reconstructed, indicating the ability of the reconstruction of real scenes with complex reflectivity distribution.

Furthermore, we demonstrate that our proposed method can achieve 3D reconstruction of a discontinuous object with variant reflectivity. We perform experimental verification by reconstructing gestures using metasurface projection. Owing to the different reflection characteristics between the human skin and background, the pattern image of the skin has some relatively rougher details, as shown in Fig. 6a. However, our algorithm implementation mostly depends on the spatial distribution feature, offering a feasible solution for the corresponding pattern search and 3D reconstruction. As expected, the depth map and 3D point cloud maps of the three gestures are recovered as shown in Fig. 6b, c, respectively, and the position, height, and orientation of the fingertips or hand are highly similar to the camera images in Fig. 6a. Eventually, both the point cloud maps of fingers (or hands) and the background are calculated successfully, indicating that our proposed 3D imaging method can achieve the reconstruction of real scenes with complex reflectivity distribution.

In addition, a discontinuous object may cause abrupt changes and large deformations of the projection pattern in camera images. Benefitting from the adaptive balance between geometry similarity and global constraint in our matching algorithm, the depth between the fingers or hands and the background was reconstructed successfully without depth fuzziness, showing the adaptability of 3D imaging for recovering discontinuous objects. Note that the results in Fig. 6 have a relatively low spatial resolution, which can be improved by increasing the number of point cloud projections.

### Spatial resolution improvement in 3D imaging

**Fig. 7:**
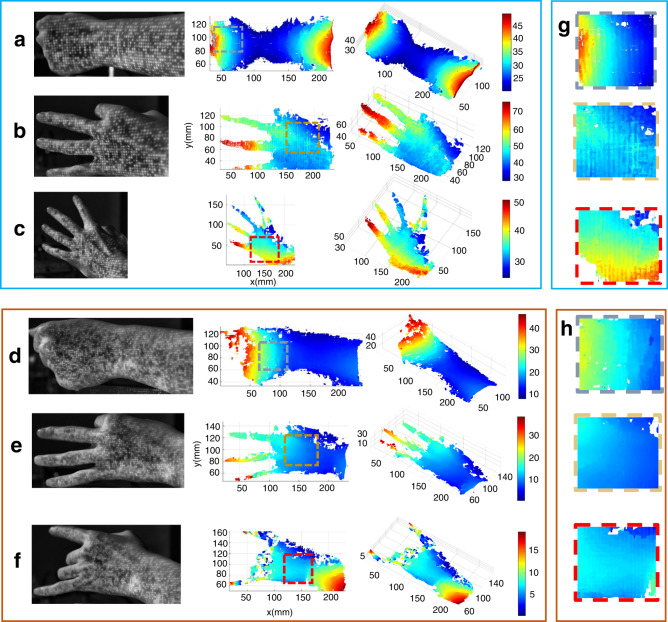
Gesture acquisition of Sample #1 (a,b,c) and Sample #2 (d,e,f). **a,b,c** 3D results of gesture with Sample #1. The captured images with Sample #1. The captured images are listed in the left column, and the top and side views of point cloud maps are shown in the middle and right columns, respectively. **d,e,f** 3D results of gesture with Sample #2. The captured images, the top and side views of point cloud maps are shown in the left,middle and right columns, respectively. **g,h** Enlarged view of the region with the boundary of the same colour as Sample #1 and Sample #2, respectively.

Because the reconstructed result with the proposed method mainly depends on the spatial features, the density of the pattern has an important effect on the spatial resolution (see Supplementary Note 9). The fundamental limitation of dot density is the pixel number of the metasurface, which will increase with the booming development of fabrication techniques (see Supplementary Note 2). We then successfully demonstrated the spatial resolution improvement with the other two samples, Sample #1 and Sample #2 (see Supplementary Note 2), in which the metasurface size was 1 mm × 1 mm, and the number of bright projection dots was 6609 and 14768, respectively. The 3D reconstructed point cloud maps of the gestures are shown in Fig. 7. Both samples can achieve 3D reconstruction, but the partial point cloud map with Sample #2 has a more continuous transition than Sample #1, as shown in Fig. 7g, h, which is caused by the denser dots endowed with smaller subsets in the match algorithm. Then, the spatial resolution improvement of 3D results will be achieved with an increasing number of dots, indicating the superiority of our method in 3D imaging.

## Discussion

A single-shot 3D imaging technique based on a metasurface is proposed in this study, leveraging the flexible light-field control ability of the metasurface and computer vision algorithms. By virtue of the frequency domain coding of Fourier holography and phase engineering of a geometry metasurface, the uniqueness of the projection pattern is generated with a single metasurface. Based on the spatial distribution of the projection pattern and detailed information regarding speckles, initial and fine match algorithms are proposed for sophisticated point cloud calculations. Moreover, a multiresolution match strategy is proposed for the fast and optimal selection of reference images, which improves the 3D accuracy and reduces the measurement uncertainty. Eventually, the capability of 3D imaging is experimentally demonstrated by cardboard deformation and gesture acquisition, indicating that the proposed method can achieve the reconstruction of textureless areas and objects with a variety of reflectance values. The combination of nanophotonics and computer vision offers the potential of a compact device for commercialisation, which may inspire further developments in 3D imaging.

In the current implementation, we used a light source and a metasurface as the projection device for 3D imaging. By integrating the metasurface with various light sources, a compact metadevice for coding projection can be created by the combination of Huygens’ metasurface and a linearly polarised laser, or using a geometric metasurface with a nano laser of circular polarisation. In addition, the metasurface possesses extra degrees of freedom for manipulating wavefronts ^[5]^, and the number of dots increases with increasing multiplexing channels (see Supplementary Note 2). The polarization conversion efficiency of our metadevice can reach to 51% at the wavelength of 820 nm experimentally.

In summary, we propose and demonstrate the use of a judiciously designed metasurface as a structured lighting module to mitigate assembly difficulties and achieve a flexible design of FOV and dots density. A corresponding reconstruction strategy and an algorithm are proposed, which demonstrate superior flexibility, robustness, and versatility. This development is significant as the requirement for equipment miniaturisation is becoming increasingly prominent with the growing demand for 3D imaging technology in consumer electronics; the results of this study may accelerate the development of applications in various domains, including computer vision, personal authentication, light detection, and artificial intelligence.

## Methods

### Phase design of the metasurface

The design strategy for a random pattern involves the use a pseudo-random binary array to produce grid locations represented by spots so that the coded pattern within an arbitrary sub-window is unique. The pattern design includes two steps: pseudorandom sequence coding and matrix construction. The phase calculation is based on the modified GS iteration algorithm. For the reconstruction observation, we use the angular spectrum propagation method with zero padding for the calculation in the Fourier plane, and the image is located in the signal region. Then, the GS iteration is performed between the hologram and Fourier planes with the constraint of the amplitude while relaxing the phase restriction to ensure high-quality point cloud images.

### Experimental setup for holographic reconstruction

A linear polariser and a quarter-wave plate are used together to guarantee the circular polarisation light to illuminate the metasurfaces. The fabricated metasurface samples are placed at the working distance of the objective lens (×40, NA = 0.6). A charge-coupled device (CCD) camera is placed at the back focal plane of a lens to capture the reconstructed holographic images in the k-space. Another pair of linear polariser and quarter-wave plate is used as the analyser to select the opposite-handedness circular polarisation light for holographic reconstruction. The experimental setup is illustrated in Supplementary Fig. S5.

### Imaging experiment of hand gestures

We ensure that the informed consent of imaging experiments of hand gestures has been obtained from all the participants.

### Reporting summary

Further information on research design is available in the Nature Portfolio Reporting Summary linked to this article.

## Supplementary information

Reporting Summary

Supplementary Information

## Data Availability

The Source data are available from the corresponding author upon request. All data needed to evaluate the conclusion are present in the manuscript and/or the Supplementary Information.

## Code availability

Codes used for this work are available from the corresponding author upon reasonable request.
