# Supplementary material for: Single-shot 3D imaging with point cloud projection based on metadevice
Source: Nat Commun. 2022 Dec 21;13:7842. doi: 10.1038/s41467-022-35483-z (PMC9772337; doi:10.1038/s41467-022-35483-z)
Supplement: Supplementary file 2 — Supplementary Information [file 41467_2022_35483_MOESM2_ESM.pdf]

Supporting information

Single-shot 3D imaging with point cloud projection based on metadevice

Xiaoli Jing[1], Ruizhe Zhao[1], Xin Li[1], Qiang Jiang[1], Chengzhi Li[1], Guangzhou Geng[2], Junjie Li[2], Yongtian Wang[1], Lingling Huang[1]*.

[1] Beijing Engineering Research Center of Mixed Reality and Advanced Display, School of Optics and Photonics, Beijing Institute of Technology, Beijing 10081, China

[2] Beijing National Laboratory for Condensed Matter Physics, Institute of Physics, Chinese Academy of Sciences, Beijing 100191, China.

Correspondence and requests for materials should be addressed to L.H. (email: huanglingling@bit.edu.cn)

Supplementary Note1: Projective transformation of structured pattern

As known, Fraunhofer diffraction can be mathematically expressed as follows.

$$E(x,y)=\frac{1}{j\lambda z}\exp\left[jk\left(z+\frac{x^2+y^2}{2z}\right)\right]\int_{-\infty}^{+\infty}\int A(\xi,\eta)\exp\left[-j\frac{k}{z}(x\xi+y\eta)\right]d\xi d\eta \tag{1}$$

where k is the wave vector and z is the distance between object A and the observation plane. (x, y) and (ξ, η) are the coordinates of the observation and object planes, respectively, and $E(x, y)$ is the electric field at the observation plane. Therefore, the intensity distribution of the plane at distance z is given by

$$I(x,y)=\left(\frac{1}{\lambda z}\right)^2\left|F\{A(\xi,\eta)\}_{f_\xi=\frac{x}{\lambda z},f_\eta=\frac{y}{\lambda z}}\right|^2=\left(\frac{1}{\lambda z}\right)^2\left|a\left(\frac{x}{\lambda z},\frac{y}{\lambda z}\right)\right|^2, \tag{2}$$

where a denotes the Fourier transform of A. The intensity distribution I_{z0}, I_{zd} at distances of z_0 and z_d should satisfy the following relationship.

$$I_{z_0}(x_0,y_0)=I_{z_d}\left(\frac{z_d}{z_0}x_0,\frac{z_d}{z_0}y_0\right) \tag{3}$$

Consequently, the pattern of the Fraunhofer diffraction region satisfies the projection transformation, as shown in the geometric relation in Supplementary Fig. 1.

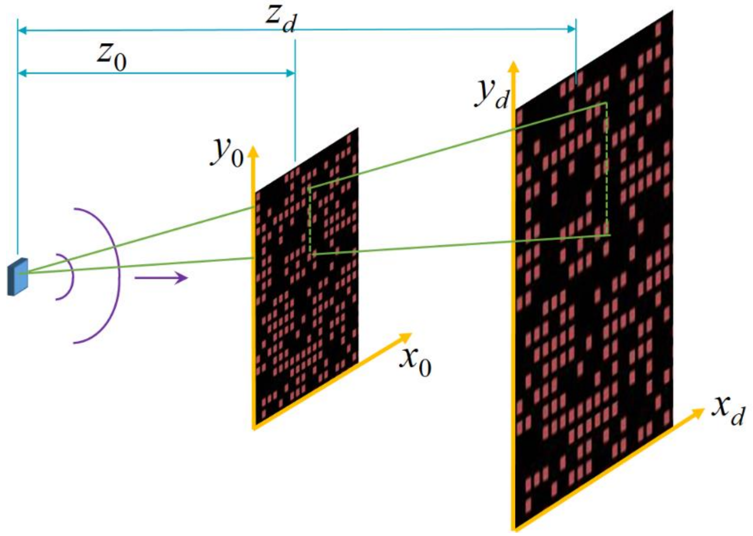

Supplementary Fig. 1 Geometry equivalence between Fourier holography and projective transform. Both planes x_0y_0 and x_dy_d are in the Fourier space, and their distances from the metasurface are z_0 and z_d, respectively.

Supplementary Note 2: Metasurface design and holographic reconstruction

Phase design. In the iterative algorithm shown in Supplementary Fig. 2, the initial phase φ_0 is applied to the target image as $a_0 = \sqrt{I_0} \bullet \exp(i\varphi_0)$, where I_0 is the intensity distribution of the target image and the resulting complex amplitude a_0 is set as the initial value of the reconstructed field a. In the iteration, the phase component Φ of the complex amplitude field of the metasurface plane calculated by the inverse fast Fourier transform (IFFT) of the reconstructed field is reserved, while the amplitude component is replaced by a uniform rectangular distribution, and the rectangular size is equal to the size of the metasurface. At the same time, the reconstructed field a is calculated using the fast Fourier transform (FFT) of the complex field A. The phase component φ is reserved, and the amplitude is replaced by the target intensity I_0. $N \times N$ is the pixel size of the metasurface, and $N_R \times N_R$ is the pixel size of the reconstructed field. $M_R \times M_R$ is the pixel size of the coded dot, and $S_R \times S_R$ is the pixel size of the coded dot spacing.

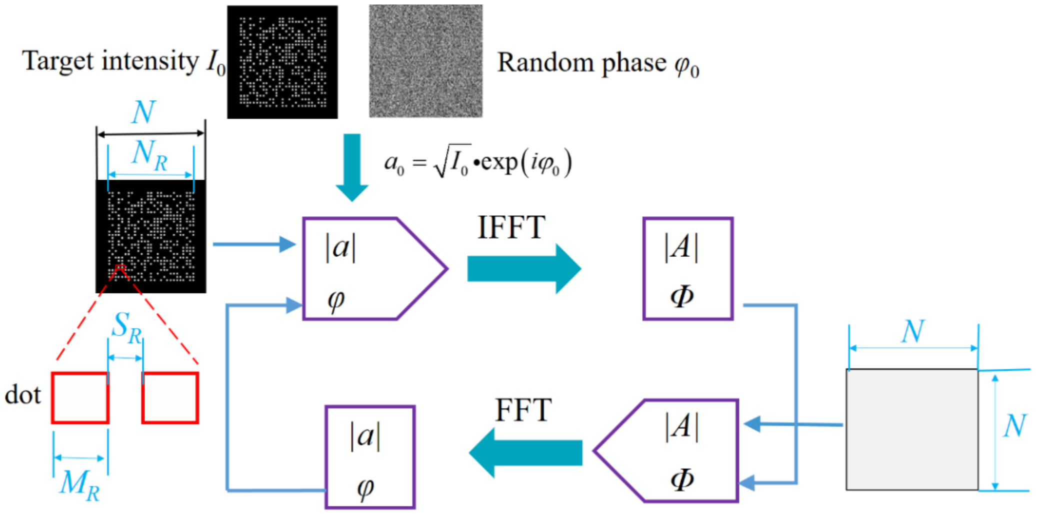

Supplementary Fig. 2 Design algorithm of phase based on the Gerchberg-Saxton (GS) algorithm.

Holographic reconstruction. In the phase-design algorithm shown in Supplementary Fig. 2, the pixel number of the target intensity is N which is the same as that of the metasurface, and the pixel number of the signal region is N_R. In addition to broadening the optimisation space, N_R is important for the field of view of the reconstruction plane. Theoretically, the FOV 2θ is determined by the maximum diffraction angle of the hologram, which is given by $\sin\theta = \lambda/2p$, where p is the pixel pitch of the metasurface. Because the period of nanopillars can be set to be smaller than half of the wavelength,

the half angle of the FOV can reach 90°, corresponding to the full space of the reconstructed field. The practical FOV can be flexibly set by adjusting the size of the reconstructed image N_R to satisfy the requirements of practical applications. The practical FOV angle $2\theta_R$ is given by $\sin\theta_R = \lambda/2p \times N_R/N$. The pixel and spacing numbers of the dots are M_R and S_R, respectively. The dot number can be calculated as $[N_R/(M_R+S_R)]^2/2$ because the bright dots account for ~50% of all coded dots. The minimal dot size is $M_R \times (\lambda z/Np)$, where z is the projection distance. We can then enhance the number of bright dots by increasing the pixel number of metasurface N and decreasing the pixel number of dot M_R and its spacing S_R. The improvement in N is related to the fabrication using a smaller period size and larger metasurface size.

We also fabricated two other samples, Sample #1 and Sample #2, and the design parameters of all the samples are listed in Supplementary Table 1. Sample #0 is the sample shown in Supplementary Fig. 3, and the dot size is obtained at a projection distance of 300 mm. Note that the diffraction pattern of Sample #1 and Sample #2 is rotational symmetric, which can be used for incident light with linearly polarization.

Supplementary Table 1 Parameter design of the reconstructed field

	Sample #0	Sample #1	Sample #2
N (pixel)	1578	3164	3164
N_R (pixel)	776	1376	1376
M_R(pixel)	8	6	4
S_R (pixel)	8	6	4
FOV $2\theta_R$ (°)	88.13	75.91	75.91
Number of bright dots	1201	6609	14768
Dot size(mm)	3.04	1.14	0.76

The characterisation of Samples #1 and #2 is shown in Supplementary Fig. 3 a, and the samples are marked with yellow and green boundaries, respectively. Supplementary Fig. 3 b gives a partial dot distribution of the target reconstructed image with the same size of 400 × 400 pixels; Sample #2 has denser dots. We also capture some images reflected from a white paper at the same distance, and the cropped images with the same

projection area are shown in Supplementary Fig. 3 c. Meanwhile, the size of the dots can be smaller than that of the dots in Sample #2 by designing a smaller pixel size M_R or larger size of metasurface $N \times p$, accompanied by a greater number of dots. We also conduct another simulation with ~26000 dots as shown in Supplementary Fig. 4. We adjust the pixel number of dot M_R and its spacing S_R to 3 pixels, while N and N_R remain constant with Samples #1 and #2; then, the number of dots is ~26000, and the minimum dot size is 0.57 mm.

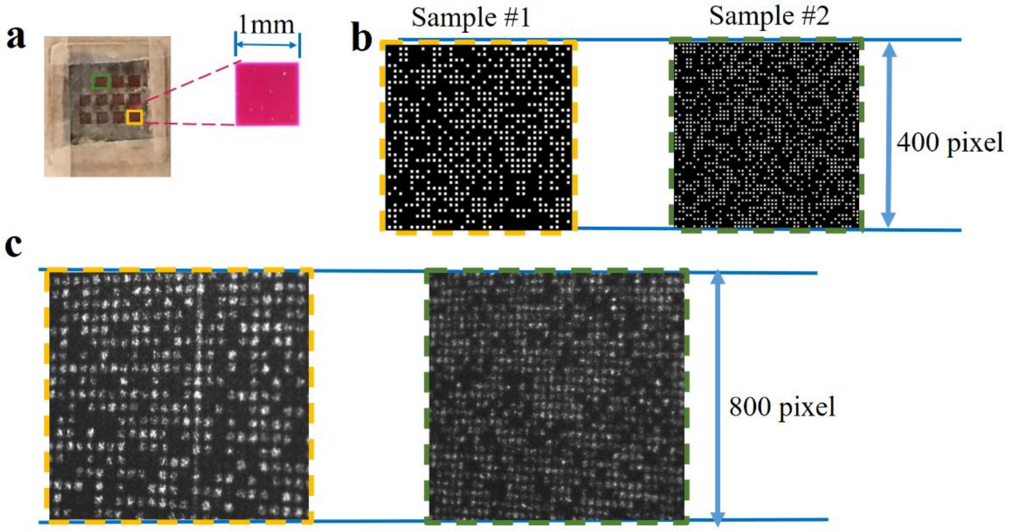

Supplementary Fig. 3 Characterisation of the metasurface with the size of 1 mm× 1 mm. **a** Two fabricated metasurfaces. Size of all metasurface samples is 1 mm × 1 mm. The boundaries of two samples are shown in yellow and green. **b** Dot density of Sample #1 and Sample #2 (partial view). Two pictures are cropped from the corresponding design pattern with the same region size. **c** Captured images reflected from white paper with the projection of two samples. The density of Sample #2 is higher, which is consistent with the design shown in **b**.

The number of dots was calculated as $[N_R/(M_R + S_R)]^2/2$. We can obtain the maximum value of $N^2/2$ when $N_R=N$, $M_R=1$, and $S_R=0$. Therefore, the number of dots is limited by the pixel number of the metasurface, which depends on the fabrication technique, including the minimum feature size and the maximum aperture size. Another method which can mitigate fabrication difficulty is polarisation or wavelength multiplexing of the metasurface; the maximum value can be described as $n \times N^2/2$, where n is the number of multiplexing channels.

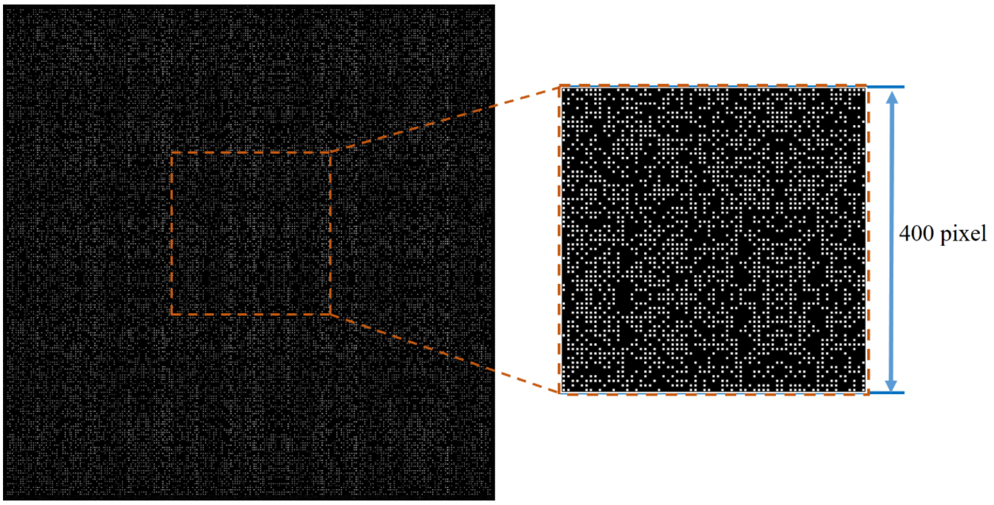

Supplementary Fig. 4 Simulated projection pattern with ~26000 dots. The size of region in the orange dotted box is 400 pixel × 400 pixel in the reconstructed holographic plane; the enlarged view is shown on the right.

To demonstrate the improvement of dots number with polarization multiplexing, we give the simulation result of polarization multiplexing as shown in Supplementary Fig. 5. By tailoring the rectangular cross-section of birefringent metasurface, it can be designed to impart distinct phases on x-linear polarized and y-linear polarized states. Hence such metasurface can generate two significantly different phase profiles for projection dots, and the reconstruction holograms are as shown in Supplementary Fig. 5 (c) and (d), respectively. Note the design parameter N_{R}, N, S_R and M_{R} are 1376, 3164, 3 and 3, respectively, which are the same as Supplementary Fig. 5. Thus we have a denser projection dots with number of ~52000 as shown in Supplementary Fig. 5 (e) when we use 45°-polarized light to illuminate the metasurface. Hence such polarization multiplexing can double the number of dots. In addition, Ref. 15 gives multichannel holographic reconstruction, which can be applied for more accurate 3D results with multi-projection and captures. Since the metasurface inherently has a higher space-bandwidth product and possesses more degrees of freedom for manipulating wavefront compared to the other conventional alternatives, the dots number increases with the increasing pixel number of metasurface and multiplexing channels.

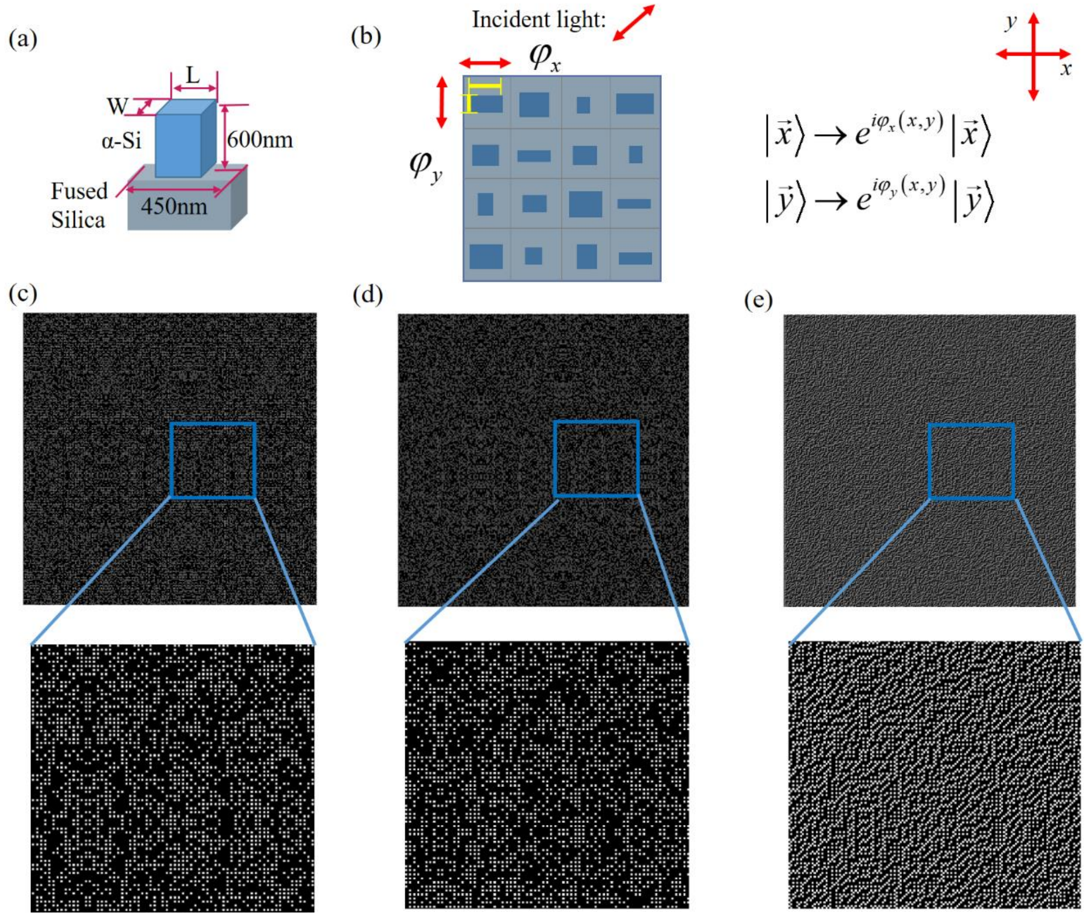

Supplementary Fig. 5 Simulation result of polarization multiplexing. (a) Birefringent metasurface for polarization multiplexing. (b) Coding phase in the two independent polarization channel. (c), (d) and (e) are the simulated reconstruction.

Efficiency analysis. Before analyzing the polarization conversion efficiency and zero-order diffraction efficiency, we briefly define these variables. Note that the zero-order diffraction efficiency discussed here is at the transmission space without the wave plate and polarizer, and the schematic is shown in Supplementary Fig. 6. The optical power passing through the bare substrate I_L as I_{in}. We measured the optical power passing through the sample I_{RL} and I_{LL}, where the subscript i and j indicate the output and input polarization components. The polarization conversion efficiency (PCE) is defined as I_{RL}/I_{in}. The zero-order diffraction energy mainly consists of the co-polarization light I_{LL}, which is caused by the discrepancy between theoretical design and fabricated sample. Therefore, the zero-order diffraction efficiency (ZDE) can be defined as,

$$ZDE = \frac{I_{LL}}{I_{in}} \tag{4}$$

And the transmission efficiency T can be calculated as,

$$T = \frac{I_{RL} + I_{LL}}{I_{in}} \tag{5}$$

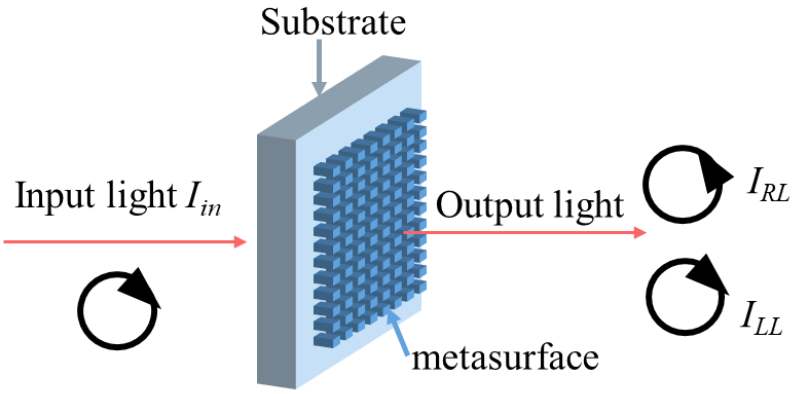

Supplementary Fig. 6 The schematic of polarization conversion efficiency and Zero-order diffraction efficiency.

Therefore, the improvement of polarization conversion efficiency can restrain the zero-order diffraction efficiency simultaneously. Note the efficiency of metasurface depends mostly on the material properties and the nanostructure design at the working wavelength theoretically. The refractive index and extinction coefficient of our used amorphous silicon (α-Si) is shown in Supplementary Fig. 7 (a). In our work, we have conducted 3D imaging validation at the wavelength of 633 nm for intuitive observation. Because the extinction coefficient k decreases with the increasing wavelength from 600 nm to 940 nm, T is relatively greater at the wavelength larger than 740 nm. The PDE and ZDE of our designed nanostructure are simulated and measured at different wavelengths ranging from 740 nm to 940 nm as shown in Supplementary Fig. 7 (b). The measured PCE and ZDE are 51% and 15% at the wavelength of 820 nm, which is much better than 20% and 28% at the wavelength of 633 nm. The measured T at the wavelength of 820 nm and 633 nm are 66%, 48%, respectively. So the fundamental limit of the efficiency is significantly better by selecting the appropriate wavelength based on α-Si. Another reason for the reduced efficiency results from manufacturing. The difference between simulated and measured data mainly results from the imperfect fabrication such as height, titled sidewall. Still, this is a subject for further investigation.

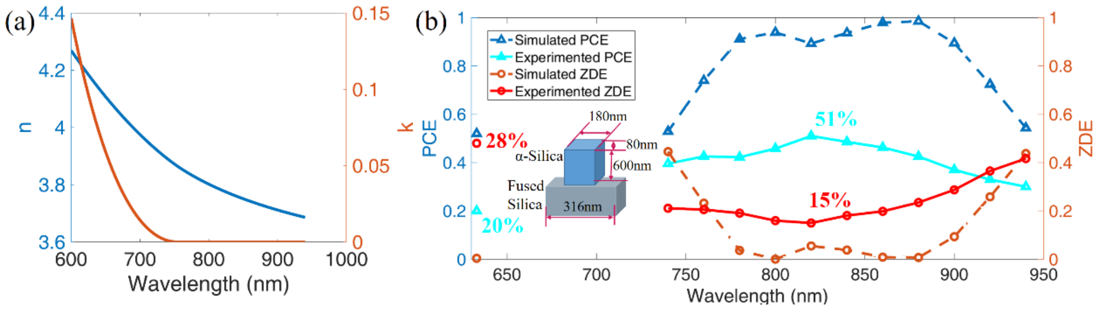

Supplementary Fig. 7 Material characteristic and efficiency analysis. (a) The refractive index *n* and extinction coefficient *k* of α-Si. (b) The simulated (Dotted line) and measured (Solid line) polarization conversion efficiency PDE and zero-order efficiency ZDE at different working wavelengths ranging from 740 nm to 940 nm. The parameter of our designed nanostructure (Figure. 2 f) is marked in the lower left corner, and the simulated and measured PDE and ZDE at the wavelength of 633 nm are also plotted for comparison.

FOV design. The upper limit of FOV equaling to 180° can be achieved benefitting from the subwavelength period size of metasurface, which is an intrinsic advantage compared with other optical elements. On this basis, we develop the 3D imaging method based on metadevice. We discuss the FOV design of projection and measured scene as follows.

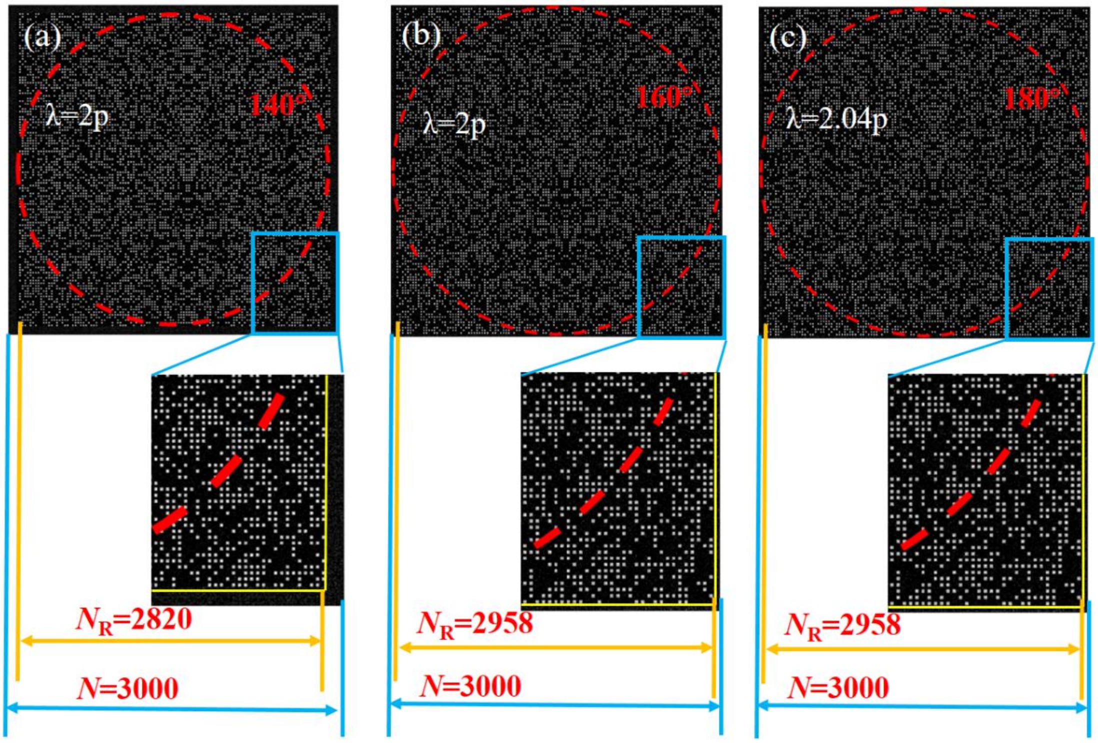

Supplementary Fig. 8 Simulation results of projection with different FOV designs. (a)

FOV > 140 °. (b) FOV > 160°. (c) FOV= 180°. The circle denotes the FOV of 140°, 160° and 180°. The value of N_R, N and the relationship of λ, p can be seen clearly in the enlarged views of two designs.

Considering the metasurface integrated with light source, we discuss the forward propagation (transmission of space) here. We develop three new simulation results with FOV of 140°, 160° and 180° by using the same method in the main text, respectively, as shown in Supplementary Fig. 8 (a-c). Hence the FOV can be indeed quite large by using metasurface compared to DOE.

Optical setup. The experimental setup for holographic image capture is shown in Supplementary Fig. 9. The setup consists of left-circularly polarised light illumination and right-circularly polarised light reconstruction. A detailed experimental description is provided in the methods section.

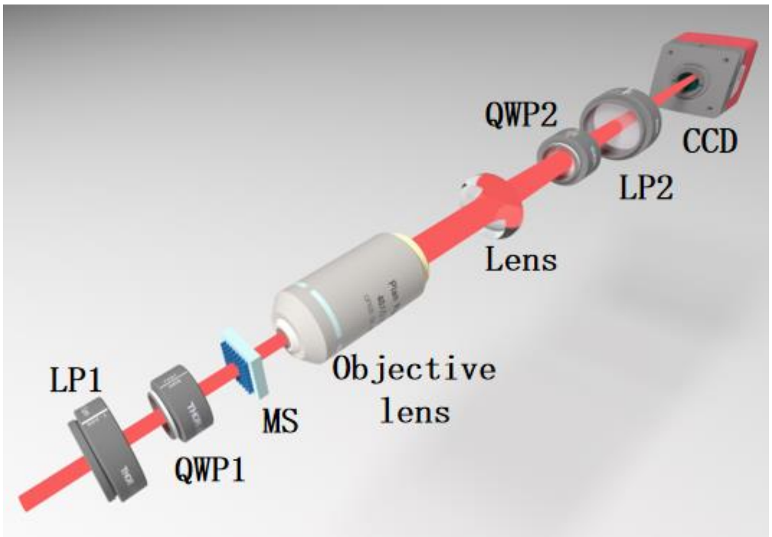

Supplementary Fig. 9 Optical setup for holographic image capture.

Supplementary Note3: Details about calibration and reconstruction of 3D imaging

To illustrate the geometric relation clearly, three points (A, B, and C) in the three planes can be used to denote the points corresponding to the same pixel of the camera, and the corresponding points of A, B, and C in the reference plane are A', B', and C', as shown in Supplementary Fig. 10 (a). Notably, A and A' are the coincide point. The point corresponding to the same pixel of the camera on an arbitrary surface is D, and the corresponding point of D in the reference plane is D'. The cross-ratio is one of the most important invariants in perspective transformation, which means that the cross-ratio of the corresponding sequence of points on a straight line remains unchanged after the geometric transformation. Based on the cross-ratio invariant and pinhole imaging model shown in Supplementary Fig. 10 (b), the relationship of the distance between *B, C, D*, and the reference plane can be calculated geometrically using P_A, P_B, P_C, and P_D.

Once the corresponding points of *A*, *B*, *C*, and *D* can be found in the image of the reference plane, *h* is determined by the known translation values H_1 and H_2. To save computing efficiency, the matching process between the points of the two auxiliary planes to the reference plane is achieved in calibration, which we call the calibration of auxiliary planes, as shown in Supplementary Fig. 10 (c). The points in the images of the auxiliary planes match well with the design pattern. As shown in Supplementary Fig. 10 (d), after matching the deformed image and the reference image, the depth *h* with the *x*th pixel can be calculated. However, the *x*-coordinates have not been calculated. Therefore, calibration using cameras is necessary. We calibrate the intrinsic parameters of the camera and the external parameters of the reference plane using Zhang's camera calibration method. Traditionally, the *x*- and *y*-coordinates have been established in the reference plane. The calibration results are shown in Supplementary Fig. 10 (e). Then, the *x* and *y* coordinate values can be obtained with the intrinsic parameter and *z* value, and the final shape of the object can be obtained completely as shown in Supplementary Fig. 10 (f).

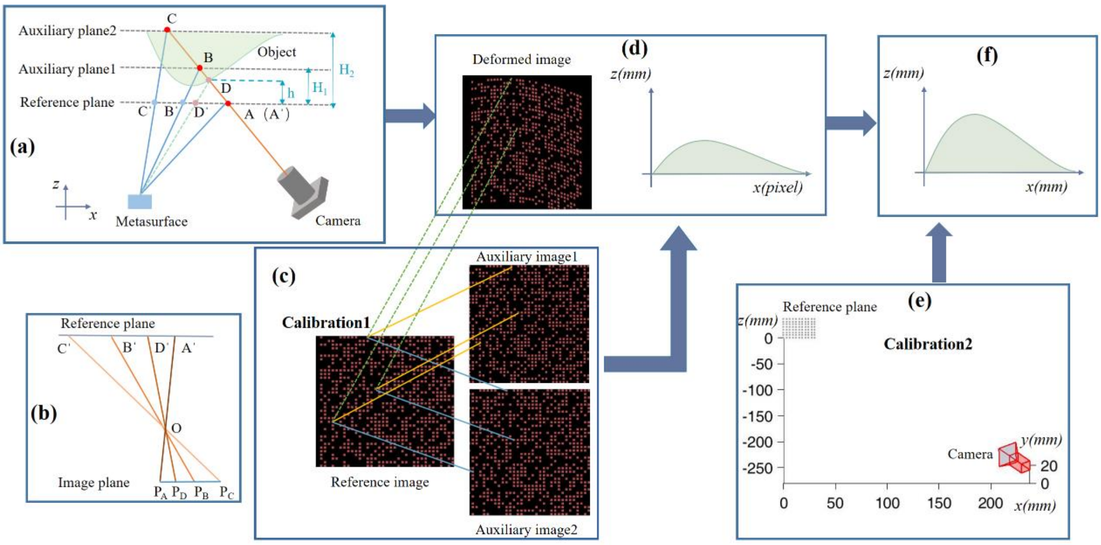

Supplementary Fig. 10 Operating principle of 3D imaging. **a** A two-dimensional diagram of triangulation. **b** Relationship between cross-ratio in reference and image planes. **c** Calibration of auxiliary planes. **d** Depth calculation of every pixel. **e** Calibration of camera. f *x*-coordinate calculation for every pixel.

A schematic of the proposed 3D imaging system is shown in Supplementary Fig. 11. In the 3D imaging experiment, the laser impinges the metasurface for pattern projection, and the camera captures the image reflected from the object. The baseline is the distance between the centre of the camera and the metasurface, and the measurement distance is approximately 300 mm in this study.

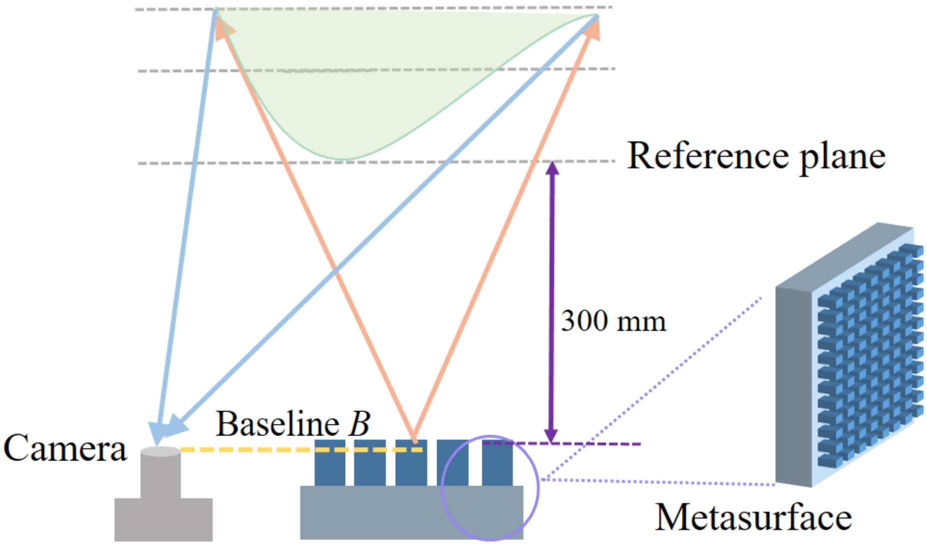

Supplementary Fig. 11 Schematic of the 3D imaging system.

Supplementary Note 4: Analysis of system calibration

In this study, the position error ranges from -0.01 to 0.01 mm, and the position range is 20 mm. H_1 and H_2 are 10 and 20 mm, respectively. We add a random position error in H_1 and H_2 and calculated the reconstructed depth h' based on the cross-ratio invariant and pinhole imaging model mentioned in Supplementary Note 2. The statistical results for the calculated relative depth error $\Delta h/h$ at depth h are shown in Supplementary Fig. 12, where $\Delta h = h' - h$, and h is the depth away from the reference plane. The mean relative depth error $\Delta h/h$ is approximately δ/H_1 (0.001) when the depth h is smaller than $2H_2$, which can be used to estimate $\Delta h/h$ in a certain depth range with a different position accuracy. Considering the relationship between the relative depth error $\Delta h/h$ and depth h, selecting the reference and auxiliary planes appropriately is an effective approach for improving the reconstruction accuracy. The proposed multiresolution search method addresses this problem.

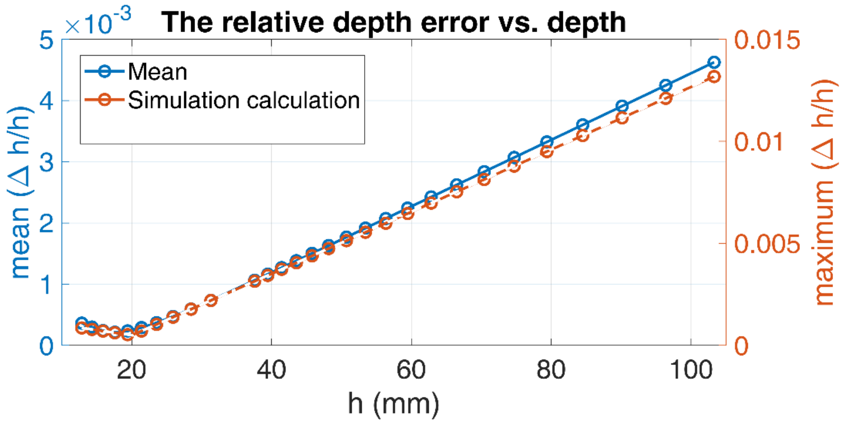

Supplementary Fig. 12. Mean relative depth error and maximum relative depth error with different depths.

Because the moving interval of the reference plane determines its selection range, a smaller moving interval can offer a reference plane for depth calculation with a smaller depth error. In our experiment, as shown in Fig. 4, the moving interval of the reference plane is 3 mm. We also conduct another experiment with a moving interval of the reference plane equalling 10 mm, as shown in Supplementary Fig. 13. The experimental setup for the accuracy assessment is shown in Supplementary Fig. 13 a. There are 10 depths in the range from 7 to 25 mm, and the reference image is at a distance of 300

mm. H_1 and H_2 are 10 and 20 mm, respectively. From Supplementary Fig. 13 b, we can see that the RMS value increases with increasing distance from the reference plane, especially when the depth from the reference plane is more than 20 mm.

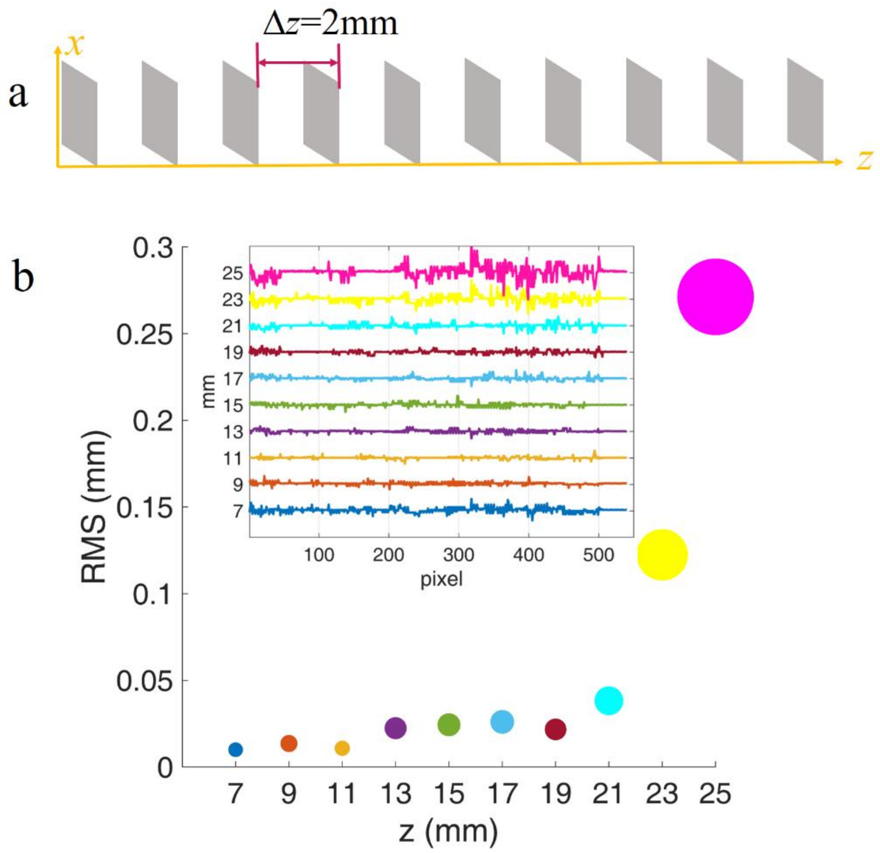

Supplementary Fig. 13. Accuracy assessment of the measurement system with moving interval of reference plane = 10 mm. **a** Measurement method. There are 10 positions to be calculated with the depth step of 2 mm, and the reference plane is 300 mm away from the metasurface. **b** RMS of each plane at different depths. The scatter size is relevant to the RMS value, and the reconstructed depth of the same image position is shown in the inset.

Supplementary Note 5: Implementation details of ZNSSD validation

To demonstrate the similarity of the speckle pattern at different depths, we calculate the ZNSSD values of nine images with different depths relative to the first image. Therefore, a small subset centred on the label is selected randomly, and the number of subsets is 18 × 20 for each image. Supplementary Fig. 14 (a–i) shows the contour maps of the corresponding ZNSSD, wherein the *x*- and *y*-coordinates denote only the calculated sequence number of labels. The results indicate that all ZNSSD values are greater than 0.9. Supplementary Fig. 14 (j) shows the statistical histograms of all the ZNSSD values in Supplementary Fig. 14 (a–i), and the maximum probability of ZNSSD is 0.92, which shows the favourable similarity of the speckle features.

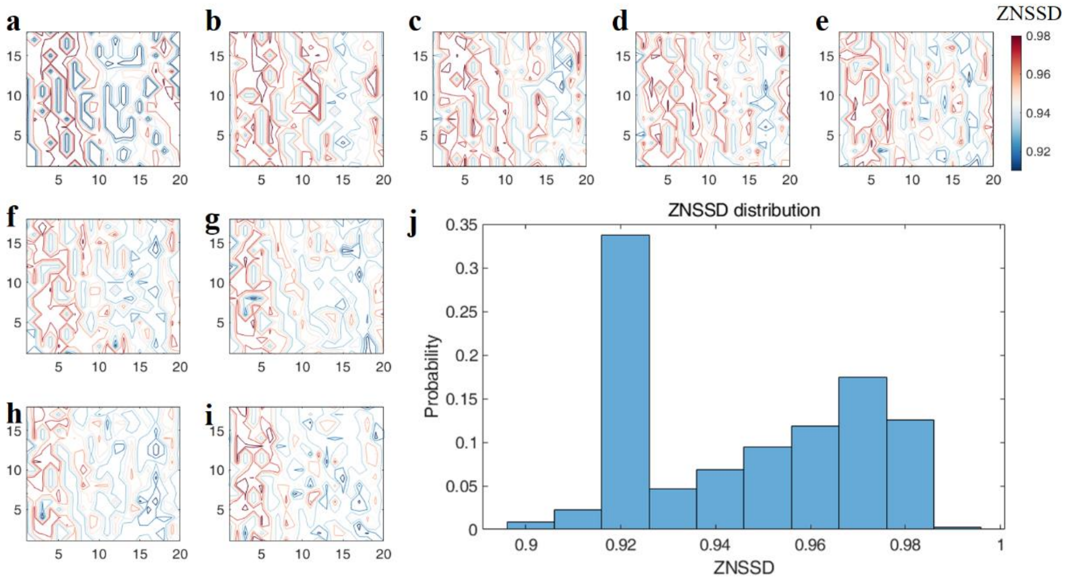

Supplementary Fig. 14 ZNSSD verification of projection pattern with different depths. **a**–**i** ZNSSD contour maps of nine images with different depths. 10 images are captured at different depths randomly and the ZNSSD calculation is conducted using the nine images with the first image. The *x* and *y* coordinates denote the serial number of randomly selected subsets of the image. The ZNSSD values are shown by the colormap. **j** ZNSSD distributions calculated by all the subsets.

Supplementary Note 6: Computational architecture of match algorithm

Our approach formulates the task of correspondence matching as a dissimilarity minimisation problem with the surface continuity constraint. First, the image is transformed into a feature domain for fast and robust matching as the initial matching process. Second, the matching task is constructed as an optimisation problem with an adaptive selection of the local region, which is a fine matching process. The computational architecture of the proposed match algorithm is shown in Supplementary Fig. 15, including the initial and the fine matches.

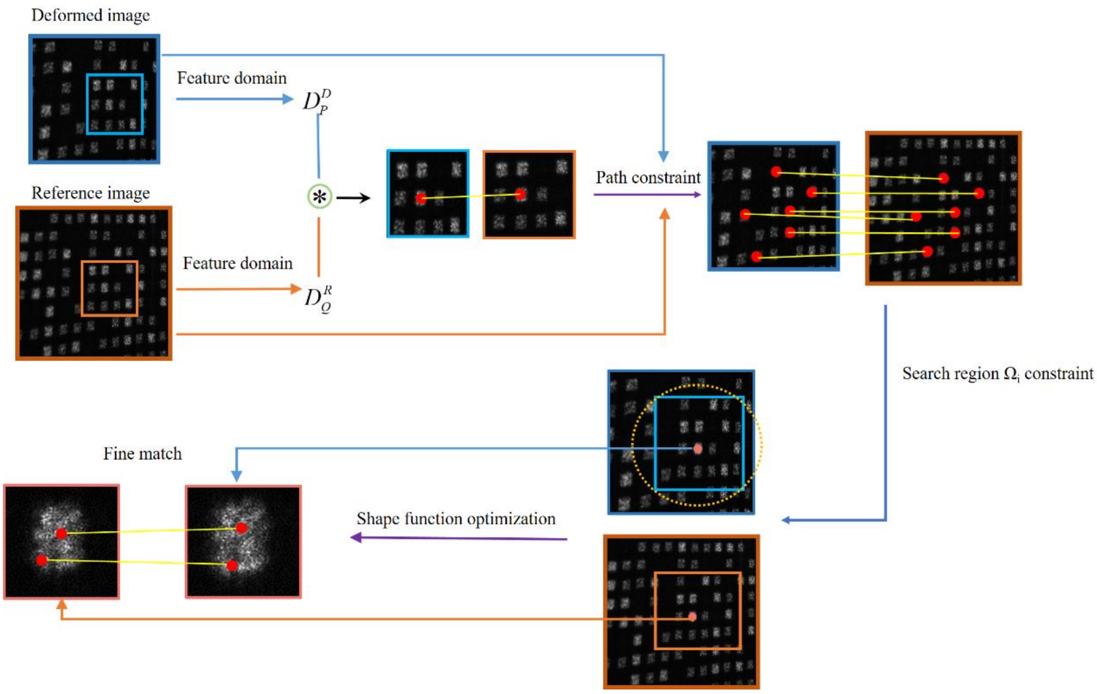

Supplementary Fig. 15 Computational architecture of match algorithm. Initial match aims to match the labels of deformed image, including feature domain transform, path guided method and similarity matching. Fine matching aims to match the inner pixel of labels based on initial match results, including adaptive subset and match optimisation based on shape function.

Computation details of initial match. Supplementary Fig. 15 describes the framework of the initial matching algorithm, which consists of three steps: First, the label is tagged for every spot in the two corresponding images using image binarization and boundary tracing. Second, the feature descriptor is utilised to describe each label of the reference and deformed images as a local descriptor of the spot distribution. Finally, the matching

process between two feature images is performed to obtain a reliable initial correspondence for all labels, including the global match and path-guided method.

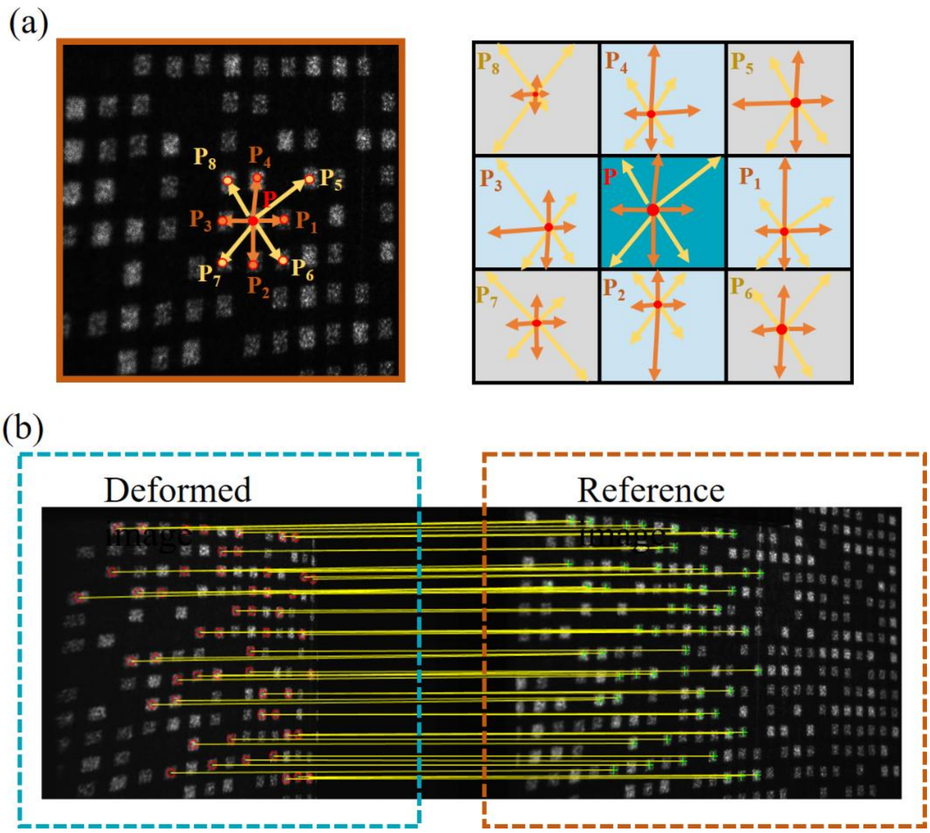

Supplementary Fig. 16 Feature descriptors and match results of initial matching. (a) Label descriptor. Low-dimensional descriptor extracted from single label and high-dimensional descriptor calculated from a certain label P and its adjacent labels P_i. The high-dimensional descriptor consists of nine weighted low-dimensional descriptors of the neighbour labels. (b) Initial matching results. The red circle and green cross denote the corresponding label in the deformed and reference images, respectively. The result of correspondence search is in good agreement with observations.

As illustrated in Supplementary Fig. 16 (a), the proposed feature descriptor is built on the distribution of neighbour labels. The red dot P represents the label that needs to be calculated, and P_i (i = 1, 2, …,8) are the neighbour labels of P, including the labels from the four main directions P_i (i = 1, 2, 3, 4) and four vice directions P_i (i = 5, 6, 7, 8). The labels in eight directions are determined by finding the nearest label in the eight equal-angle parts, and the low-dimensional label descriptor can be computed according

to Supplementary Equation (6):

$$V_P = [P_1P; P_2P; \cdots P_8P] \tag{6}$$

where P_1P is the distance between labels P_1 and P, etc. The low-dimensional descriptor of label P is shown in Supplementary Fig. 16 (a). The vector is then further normalised into a unit norm as V_P. Obviously, the label descriptor V_P can cope well with the scaling, translation, and illumination variation issues encountered in a real scene, considering that V_P is related only to the relative distance between adjacent labels and label P. The discriminative label distributions in different directions around each label make their corresponding feature descriptors more distinctive from one another, which is helpful for matching two corresponding labels between the reference image and the deformed image. To improve the matching robustness, a more elaborate descriptor D_P is formed from the low-dimensional label descriptor of each adjacent label using the following equation:

$$D_p = \left[V_P; \omega_1 V_{P1}; \omega_1 V_{P2}; \omega_1 V_{P3}; \omega_1 V_{P4}; \omega_2 V_{P5}; \omega_2 V_{P6}; \omega_2 V_{P7}; \omega_2 V_{P8}\right] \tag{7}$$

where ω_1 and ω_2 are the weight values of the main and vice directions, and their values are set to 0.6065 and 0.3679, respectively, in analogy with a Gaussian kernel playing a role in the adjacent region of the centre label. Similar to the scale-invariant feature transform (SIFT) feature descriptor, our feature descriptor consists of 72 values from 25 labels and eight orientations, and its performance is similar to SIFT in many respects, including robustness to lighting, blur, and perspective distortion. Meanwhile, our proposed method is more capable of managing sparser features than SIFT. Thus, the cosine distance between the target label descriptor D_P^D and reference label descriptor D_Q^R can be used to evaluate the similarity according to Supplementary Equation (8):

$$CD_{PQ} = \frac{D_P{}^D * D_Q{}^R}{\left|D_P{}^D\right| * \left|D_Q{}^R\right|} \tag{8}$$

where $\left|D_P^D\right|$ and $\left|D_Q^R\right|$ are the norms of the descriptor vectors D_P^D and D_Q^R, respectively. * denotes the dot product. A large cosine distance indicates high similarity, which can

be used to perform a similarity match. The feature domain transform can describe $f(x, y)$ and $g(x, y)$ as D^D and D^R, respectively, which are mathematical sets of vectors, as shown in Supplementary Fig. 16 (a). Therefore, during the initial match, Supplementary Equation (4) can be written as follows.

$$\begin{aligned} &\arg\min \left\| D^D_{l_i^D} - D^R_{C_i} \right\|_2^2 \quad where\ l_i^D \subset U^D \\ &\text{st.} \quad l_i^{D,k+1} = F_c(\Gamma_i^{D,1}, \Gamma_i^{D,2}, \cdots \Gamma_i^{D,k}) \end{aligned} \tag{9}$$

where i is the index of the labels, C_i is the corresponding label of label l_i^D, k is the number of processed labels, and $l_i^{D,k+1}$ is the (k+1)th label that needs to be processed. F_c denotes the matching strategy based on surface continuity, including global matching and path-guided methods.

Global matching aims to search the faithfully matching label in the reference label based on feature descriptors. It is a very robust method and is used to find the corresponding label of the first calculated label in the entire reference image. The global match algorithm includes a similarity search based on cosine distance, as mentioned above, and a forward-backward consistency check. The forward–backward consistency check consists of two similarity matching operations, in which forward matching is performed by calculating the cosine distance between all reference labels and label $l_i^{D,1}$ and finding the corresponding label C_i^1 based on the maximum cosine distance, and backward matching is performed by finding the corresponding label $l_i^{D,1\prime}$ of C_i^1 in the deformed labels. Therefore, the consistency of labels $l_i^{D,1\prime}$ and $l_i^{D,1}$ can ensure the global optimum of the corresponding label search of the first label, leveraging the spatial uniqueness of the pattern design. A forward-backward consistency check is necessary, which can also be used in isolated label matching and does not require too much time for the entire computation process because of the limited loops of calculation.

The next label that needs to be processed can be found by the adjacent label distribution, which we call the path-guided method. The path-guided method aims to make the match process always start from the labels with high density and accurate adjacent corresponding labels; then, label $l_i^{D,k+1}$ should be determined from the adjacent labels Γ of processed labels combined with neighbour information capacity. If label

$l_i^{D,k+1}$ is not found, which means that weak connectivity occurs in the processed and unprocessed labels, the global match algorithm is used to find label $l_i^{D,k+1}$. To guarantee the accuracy of the match results, a similarity check based on cosine distance calculation should be performed in each match to remove the wrong label and switch to another path to prevent error propagation. The initial matching results for the labels are shown in Supplementary Fig. 16 (b).

Computational architecture of fine match. The fine match algorithm aims to achieve a subpixel match using the grey details of the inner labels, as shown in Supplementary Fig. 17 (a). The inverse-compositional Gauss-Newton (IC-GN) algorithm is used here, which is a popular subpixel registration algorithm widely used in the digital image correlation (DIC) technique. Owing to the limited convergence range of the traditional IC-GN algorithm, an improved algorithm is proposed, which is well adjusted for fine matching in a metasurface projection. For completeness, we discuss the derivation of the IC-GN algorithm with a second-order shape function in the paragraphs below.

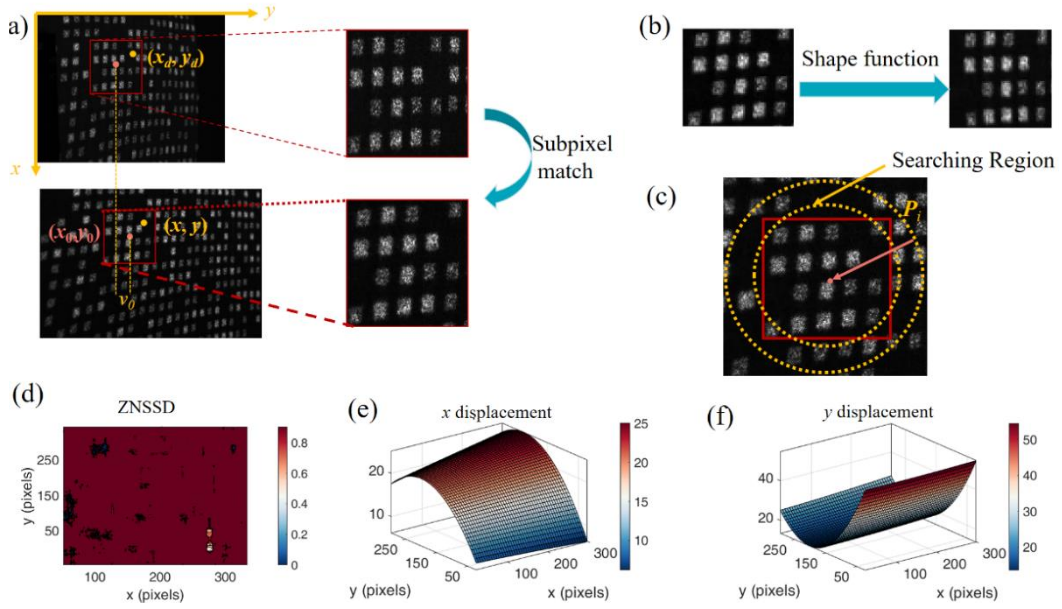

Supplementary Fig. 17 Computational details and match results of fine match. (a) Definition of two image coordinates. (x_d, y_d) and (x_0, y_0) are the coordinates of the subset centre, and (x, y) is the coordinate of the point in the subset. (b) Transformation of images based on shape function. (c) Algorithm diagram of adaptive subset. The searching region keeps the red centre point constant and the region size diminishing

shown as the yellow dotted line. (d) ZNSSD values after fine match. (e) and (f) are the displacement between deformed image and reference image along x-direction and y-direction, respectively.

The IC-GN algorithm aims to achieve sub-pixel matching accuracy by modelling the match process as a nonlinear optimisation problem with a shape function. The shape function can be used to describe the deformation characteristics of a local coordinate system. Because a higher-order shape function can be used to describe more complicated deformations, a second-order shape function is used here, which is widely used in DIC. As shown in Supplementary Fig. 17 (a), the mathematical relationship between point (x_d, y_d) in the deformed image and point (x, y) in the reference image can be described as follows:

$$\begin{aligned} x_d &= x_0 + u_0 + u_x\Delta x + u_y\Delta y + \frac{1}{2}u_{xx}\Delta x^2 + \frac{1}{2}u_{yy}\Delta y^2 + u_{xy}\Delta x\Delta y \\ y_d &= y_0 + v_0 + v_x\Delta x + v_y\Delta y + \frac{1}{2}v_{xx}\Delta x^2 + \frac{1}{2}v_{yy}\Delta y^2 + v_{xy}\Delta x\Delta y \end{aligned} \tag{10}$$

where $\Delta x = x - x_0$ and $\Delta y = y - y_0$, and (x_0, y_0) represent the coordinate values of the subset centre, which is shown as a red point in Supplementary Fig. 17 (a). u_0, v_0 are the displacements of the centre label along the x- and y-directions, respectively. u_x, u_y, v_x, v_y are the first-order parameters, and u_{xx}, u_{yy}, u_{xy}, v_{xy}, v_{yy}, v_{xy} are the second-order parameters. Thus, **p** is the parameter vector of the shape function, as shown in Supplementary Equation (11).

$$\mathbf{p} = (u_0, u_x, u_y, u_{xx}, u_{xy}, u_{yy}, v_0, v_x, v_y, v_{xx}, v_{xy}, v_{yy})^T \tag{11}$$

The ZNSSD criterion can be described as

$$C_{ZNSSD}(\Delta\mathbf{p}) = \sum_{x=-M}^{M}\sum_{y=-N}^{N}\left\{\frac{f(W(x,y;\Delta\mathbf{p})) - f_m}{f_s} - \frac{g(W(x,y;\mathbf{p})) - g_m}{g_s}\right\}^2 \tag{12}$$

where Δ**p** is the increment of **p.** f_s, g_s are calculated as below:

$$\begin{aligned} f_s &= \sqrt{\sum_{x=-M}^{M}\sum_{x=-N}^{N}\left[f(W(x,y;\Delta\mathbf{p})) - f_m\right]^2} \\ g_s &= \sqrt{\sum_{x=-M}^{M}\sum_{x=-N}^{N}\left[g(W(x,y;\mathbf{p})) - g_m\right]^2} \end{aligned} \tag{13}$$

To find the extrema of C_{ZNSSD}, $\Delta\mathbf{p}$ can be deduced as follows:

$$\Delta\mathbf{p} = -\boldsymbol{H}^{-1}\sum_{x=-M}^{M}\sum_{y=-N}^{N}\left\{\left(\nabla f\frac{\partial W}{\partial \mathbf{p}}\right)\left[f\left(\boldsymbol{W}\left(x,y:0\right)\right)-\bar{f}-\frac{f_s}{g_s}g\left(\boldsymbol{W}\left(x,y:\mathbf{p}\right)\right)+\frac{f_s}{g_s}\bar{g}\right]\right\} \tag{14}$$

where $\boldsymbol{H}$ is the Hessian matrix of reference subset:

$$\boldsymbol{H} = \sum_{x=-M}^{M}\sum_{y=-N}^{N}\left\{\left(\nabla f\frac{\partial \boldsymbol{W}}{\partial \mathbf{p}}\right)^{T}\left(\nabla f\frac{\partial \boldsymbol{W}}{\partial \mathbf{p}}\right)\right\} \tag{15}$$

Then, one can update the shape function $\boldsymbol{W}$ (x, y; $\boldsymbol{p}$) until $\Delta\mathbf{p}$ reaches the pre-set threshold.

Based on this, the adaptive subset size and initial value of the shape function, which utilise the initial match result C as a global constraint, are proposed for accurate matching. Undoubtedly, the initial guess of $\mathbf{p}$ plays a pivotal role in the iterative IC-GN algorithm, and a reliable initial guess guarantees that the IC-GN can converge quickly at the desired value. Therefore, the initial match result C can be used to estimate the initial guess of the IC-GN algorithm. Because the second-order shape function has 12 deformation parameters, at least six pairs of label matching need to be calculated for the initial estimation. The shape transform based on the initial matching results is shown in Supplementary Fig. 17 (b). To achieve a more sophisticated match of the local subset, we set an optimisation function as in Supplementary Equation (16).

$$\min\left\| f\left(x,y\right)*\Omega_i\left(x,y\right)-g\left(W_i\left(x,y;\mathbf{p}\right)\right)*\Omega_i\left(W_i\left(x,y;\mathbf{p}\right)\right)\right\|_2^2 \tag{16}$$

where $\Omega_i\left(x,y\right)$ is the subset function with values 0 and 1. This aims to minimise the difference between the target and reference subsets by adaptive selection of subset clustering based on the initial match results, which is used to constrain the global continuity at the same time. First, an approximate size of the search region is given as a yellow circle with a larger radius, as shown in Supplementary Fig. 17 (c), and the label pairs in the search region are used to estimate the initial deformation vector $\mathbf{p}$ using the least-squares method. To eliminate the disturbance caused by inaccurate and false label matching, labels with large residual errors are discarded if they are not within the boundary of the search region. Subsequently, $\mathbf{p}$ is calculated using valid labels.

Second, the initial deformation vectors $\mathbf{p}_i$ are calculated using the diminishing search region along the radial direction, where $\mathbf{p}_i$ is the vector $\mathbf{p}$ calculated at the ith search region. Then, the maximum region that meets the requirement is chosen such that the variation of $\mathbf{p}_i$ is less than the pre-set threshold. Finally, the region and the corresponding vector $\mathbf{p}_i$ are used to achieve IC-GN algorithm, and the ZNSSD coefficient, x-displacement and y-displacement after fine match are shown in Supplementary Fig. 17 (d), (e) and (f), respectively. Consequently, most ZNSSD coefficients are larger than 0.85, indicating a good match.

Supplementary Note 7: Matching accuracy with subset size

The window size used in the metasurface design determines the minimum distinctive region for matching the label; hence, we design the feature descriptor with an equivalent window measuring 5 label × 5 label, which is larger than the window size of the metasurface design which measures 4 label × 4 label. After label matching, the subset size used in the fine matching algorithm can be set to be smaller than the window size used in the metasurface design. It is well known that the depth accuracy increases (depth deviation decreases) with increasing subset size. Combined with our adaptive subset selection of the fine match algorithm, a match accuracy of ~0.3 pixel is achieved. A detailed estimation of the depth accuracy of different subset sizes with three samples is discussed below.

Because the matching accuracy is related to the image noise and the sum of squares of subset intensity gradients (SSSIG), where the image noise is an approximate and statistical value, a simulation based on the real images captured by the camera and numerical deformations is conducted for quantitative analysis. Numerical deformations are applied to the captured pattern images using cubic interpolation. In the simulation, the displacement along the y direction is 0.5 pixel, and the subset size of the fine match algorithm are set to 31 × 31, 51 × 51, 71 × 71, 91 × 91, 111 × 111, 131 × 131, 151 × 151, 171 × 171, 191 × 191, and 211 × 211 pixels, respectively. The standard deviation and maximum deviation are used to quantitatively evaluate the calculated displacements, and are defined as,

$$std\_v_e = \sqrt{\frac{1}{N-1}\sum_{i=1}^{N}\left(v_{mean} - v_{real}\right)^2} \tag{17}$$

$$max\_v_e = \max\left(abs\left(v - v_{real}\right)\right) \tag{18}$$

where v and v_{real} are the calculated and real displacements, respectively, and the bias error is the difference between v and v_{real}. std_v_e and max_v_e are the standard deviation and maximum deviation of all calculated points, respectively, and the number of calculated points N is 12635. The deviation results for three samples of different subset sizes are shown in Supplementary Fig. 18. The standard deviation and maximum

deviation decrease as the subset size increases, and the deviation decreases slowly when the subset size is larger than 91×91 pixels for Sample #0 and 51×51 pixels for samples #1 and #2. Deviation decreases with increasing density of dots, and Sample #2 has the smallest deviation under all subset sizes. Density improvement is important for the metasurface projector, which can make the calculation more accurate and faster by selecting a small window. Here, we empirically consider $6\sigma_v$ as the match error (match uncertainty). We have checked our adaptive subset size in the depth measurement, and the size is mostly distributed in the range from 81 to 111 for Sample #0, 61 to 81 for Sample #1, and 31 to 61 for Sample #2. The matching errors for the three samples can then be determined as approximately 0.26, 0.24, and 0.26 pixel approximately.

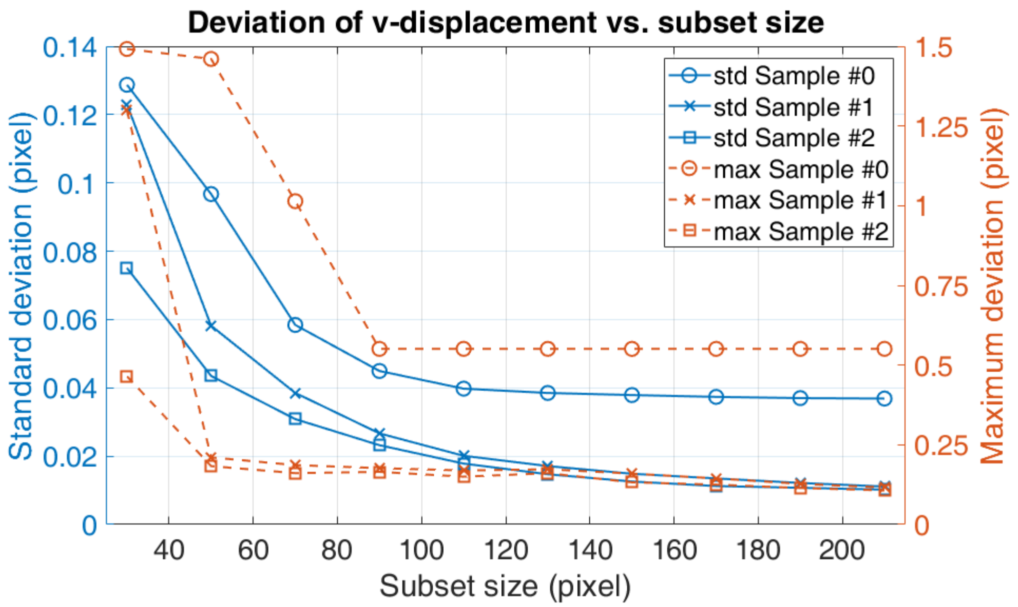

Supplementary Fig. 18 Deviation results with different subset size of Sample #0, Sample #1, and Sample #2. Different samples are shown with different markers.

Supplementary Note 8: Accuracy improvement with multi-resolution search strategy

We experimentally demonstrate the effectiveness of the multiresolution search method. Specifically, the reference images are captured with an interval of 2 mm along the z-direction in Fig. 4 (a), and two low-resolution images and one original image are used to achieve a multi-resolution search. Simultaneously, a reference image of $z = 0$ mm is used to calculate the depth map, and the results are shown in Supplementary Fig. 19. Compared with Fig. 4 (a), the multiresolution search method can achieve a smoother depth map rather than a fluctuant depth map caused by depth uncertainty. PV and RMS are used to evaluate the planeness of the test planes, and an enlarged view of the fluctuant point cloud is shown in Supplementary Fig. 19 (b). The maximum PV and RMS values are almost 3.2 and 0.12 mm, respectively, because of the fluctuant data and boundary noise. However, the maximum of PV and RMS value in Fig. 4 (b)–(c) are 0.24 mm and 4.4×10^{-4} mm, which are 7.8% and 0.37% of the results with fixed reference images. Consequently, the proposed search strategy shows a significant advantage in the elimination of noise and uncertainty in point cloud data. Meanwhile, the step values are calculated with the difference in the mean depth of the two planes in each measurement setup, and the difference with the real depths is shown in Supplementary Fig. 19 (d). The maximum step error is 0.2 mm; this value is relatively similar to the results obtained by multi-resolution search method. It should be noted that the data fluctuation is mainly attributed to the fuzzy similarity, but the mean value eliminates the error caused by fluctuation, leading to similar results as the multi-resolution search method. Therefore, reconstruction with fixed reference images is suitable for 3D positioning with similar results and relatively lower computation cost, while reconstruction with a multiresolution search strategy performs better for 3D imaging.

Another instruction regarding depth accuracy is that the proposed method for 3D imaging is based on triangulation, which can have better accuracy with a large baseline and focal length of the camera. Furthermore, the appropriate adjustment of the system parameter can yield a satisfactory result with our proposed method, showing the

flexibility and versatility for different scenes with different accuracy requirements.

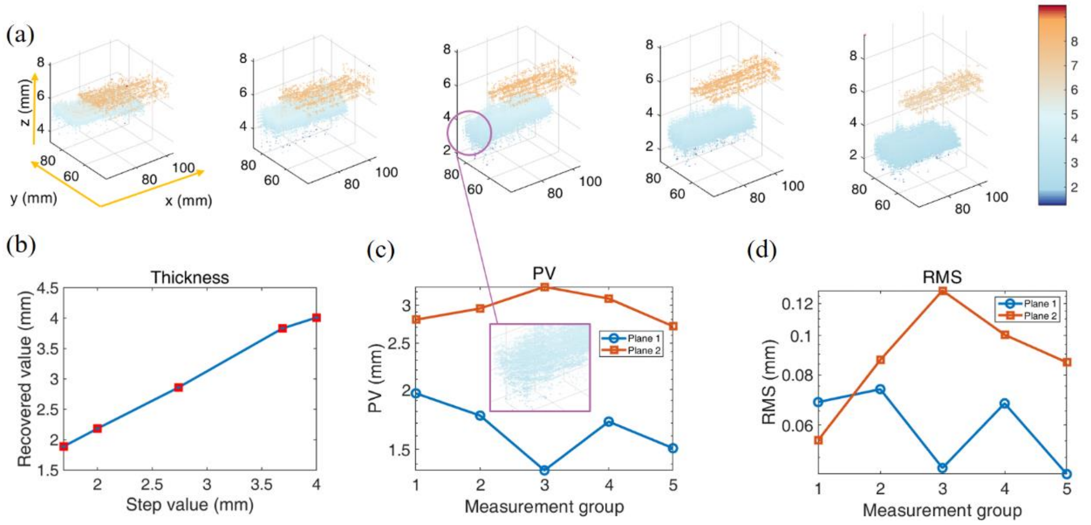

Supplementary Fig. 19 Results of the same setups of Fig. 4 calculated with fixed reference images. (a) Recovered point cloud images with five different height differences. The data fluctuation of point cloud seems more serious compared with the results in Fig. 4, and a local point cloud data is shown in the enlarged view of the third setup. (b) The recovered thickness with real values and the thickness error with five setups is 0.2,0.18,0.12,0.14, and 0.09 mm, respectively. (c)–(d) The PV and RMS values of the planes of five setups. The legend of plane 1 represents the higher plane in measurement setups and plane 2 represents another. The maximum PV and RMS values are 3.3 and 0.13 mm, respectively.

Computation speed. The computation speed mainly depends on the accuracy requirement, data volume, hardware platform and algorithm design. The computation cost mainly depends on the subpixel search in the IC-GN algorithm, including the initial deformation parameter **p**, the order of the shape function (second order), the size of the selected subset, the interpolation calculation (bicubic interpolation) in each subset and the iteration times in the nonlinear optimization. Considering another key performance of the algorithm as accuracy, the order of the shape function and the interpolation method provide a more accurate result with more calculation time. Hence we discuss the acceleration method of our proposed algorithm while keep the two point mentioned above invariant for fairness. Since the coarse match give a reliable initial parameter p, the quick convergence of the algorithm can be achieved. Hence we revise our algorithm

design for avoiding the redundant computation. In each iteration, the updated g ($\boldsymbol{W}$ (x, y; $\mathbf{p}$)) needs to be interpolated with the new deformation $\mathbf{p}$. The bicubic interpolation is implemented to determine the gray values at subpixel locations as follows.

$$g(x,y)=\sum_{m=0}^{3}\sum_{n=0}^{3}a_{mn}x^{m}y^{n} \tag{19}$$

where a_{mn} is the interpolation coefficients determined by the gray intensities of the neighboring 4×4 pixels, which is fixed. So the interpolation coefficients a_{mn} can be designed as a look-up table, which needs to be calculated only once rather than all the iteration numbers of the participant calculated subsets. Therefore, we can achieve the reconstruction of 100×100 spatial size data in 1.2s with Sample #2 by using multi-resolution strategy, and 0.9 s with the fixed reference plane. Note that the test platform uses i7-8700 CPU with 3.19 GHz main frequency under Matlab 2021a configuration.

Supplementary Note 9: Discussion on spatial resolution and depth resolution

Before analysing the spatial and depth resolutions, we briefly define these variables. To simplify the discussion, we use the following definitions, considering the effects of the system and algorithm parameters.

1). The spatial resolution is defined as the minimum distinguishable distance in the x-y plane.

2). The depth resolution is defined as the minimum distinguishable distance along the z-direction.

Although the improvement of spatial resolution intuitively indicates a smaller size (whether in physical units or pixels), the term "decreasing the spatial resolution" indicates a smaller distance.

Analysis of the spatial resolution. A spatial averaging effect occurs in the reconstructed point cloud, as shown in Fig. 6, which is a rather intuitive phenomenon. This is caused by the nature of the IC-GN algorithm in our fine match, which is similar to a low-pass filter. We explain the spatial resolution according to the Savitzky-Golay (SG) low-pass filter in a one-dimensional form. The amplitude attenuation with different subset sizes is shown in Supplementary Fig. 20 and the normalised wave number $\tilde{k}$ ranges from 0 to 1, where the maximum practical wave number is equal to the Nyquist sampling frequency of 1/2. A higher cut-off frequency occurs with a small subset, which corresponds to a small spatial resolution. For quantitative analysis of the spatial resolution, we use the corresponding normalised wave number k with an amplitude of 0.7 to evaluate the spatial resolution σ_{xy}, which is given by $\sigma_{xy}=2/k$. Thus, the spatial resolutions σ_{xy} with subset sizes of 21, 31, 41, 51, 61, and 101 pixel are 18, 27, 38, 44, 57, and 95 pixel, respectively. Note that the spatial resolution σ_{xy} determined above is not the exact value because the amplitude is selected empirically.

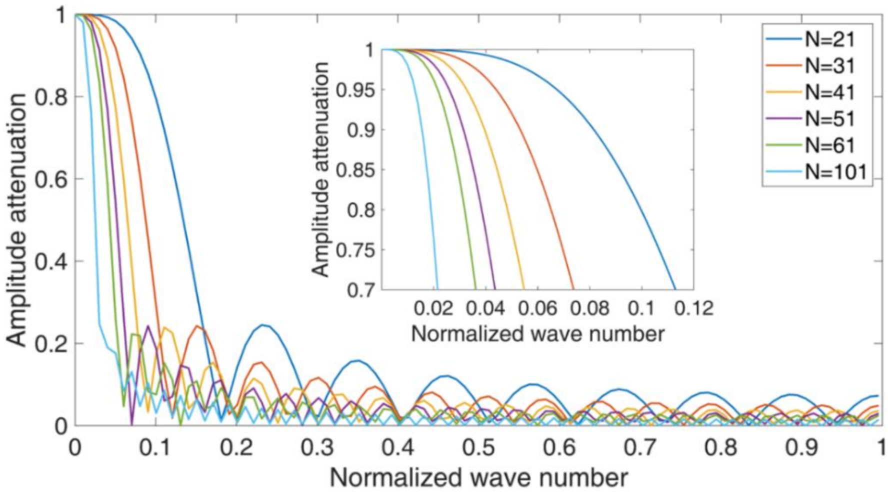

Supplementary Fig. 20 Amplitude attenuation with different subset size. We simulate the transfer function in a one-dimensional form, and the subset sizes N are 21, 31, 41, 51, 61, and 101 pixels, respectively. The inset is the enlarged view of the amplitude with the normalised wave number of 0–0.2.

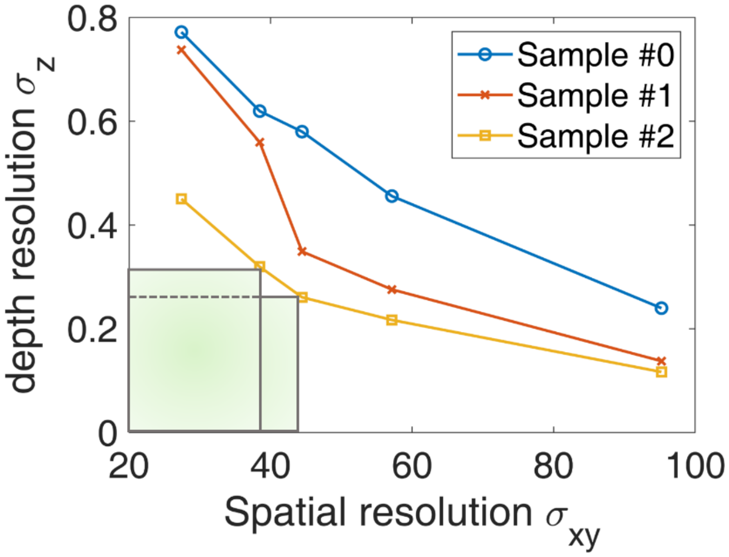

Supplementary Fig. 21 The space of depth resolution and spatial resolution with different samples. The subset size is 31 × 31, 41 × 41, 51 × 51, 61 × 61, and 101 ×101 pixels, respectively. The green rectangular region indicates the region constructed by the depth and spatial resolutions.

Trade-off between the spatial resolution and depth resolution. Combined with the results in Supplementary Note 7, we can provide the depth resolution σ_z of the three samples with different subset sizes, as shown in Supplementary Fig. 21. The spatial resolution σ_{xy} increases with increasing subset size, whereas the depth resolution

decreases. We then evaluate the resolution space with the product of depth resolution and spatial resolution in analogy with the space-bandwidth product, where a smaller product value indicates a good resolution space. As we can see, the product is the area of the rectangular region enclosed by the coordinate axes, and Sample #2 always has the smallest product. Because the density of Sample #2 is the largest, the depth resolution σ_z is the smallest for the same subset size as the other samples. In addition to the selection of the subset size, the dot density of the projection is the fundamental limit of the spatial resolution and depth resolution, which indicates the information capacity of illumination. The 3D imaging experiments of gesture recognition with Samples #1 and #2 in Fig. 7 also support this conclusion.

Supplementary Note 10: 3D reconstruction with a larger object size

The schematic of 3D reconstruction is shown in Supplementary Fig. 22 (a). The 3D reconstruction can be achieved in the entire projection FOV, and an experiment of the objects with larger size has conducted as shown in Supplementary Fig. 22 (b) and (c), which the FOV of measured scene is ~80°× 69°.

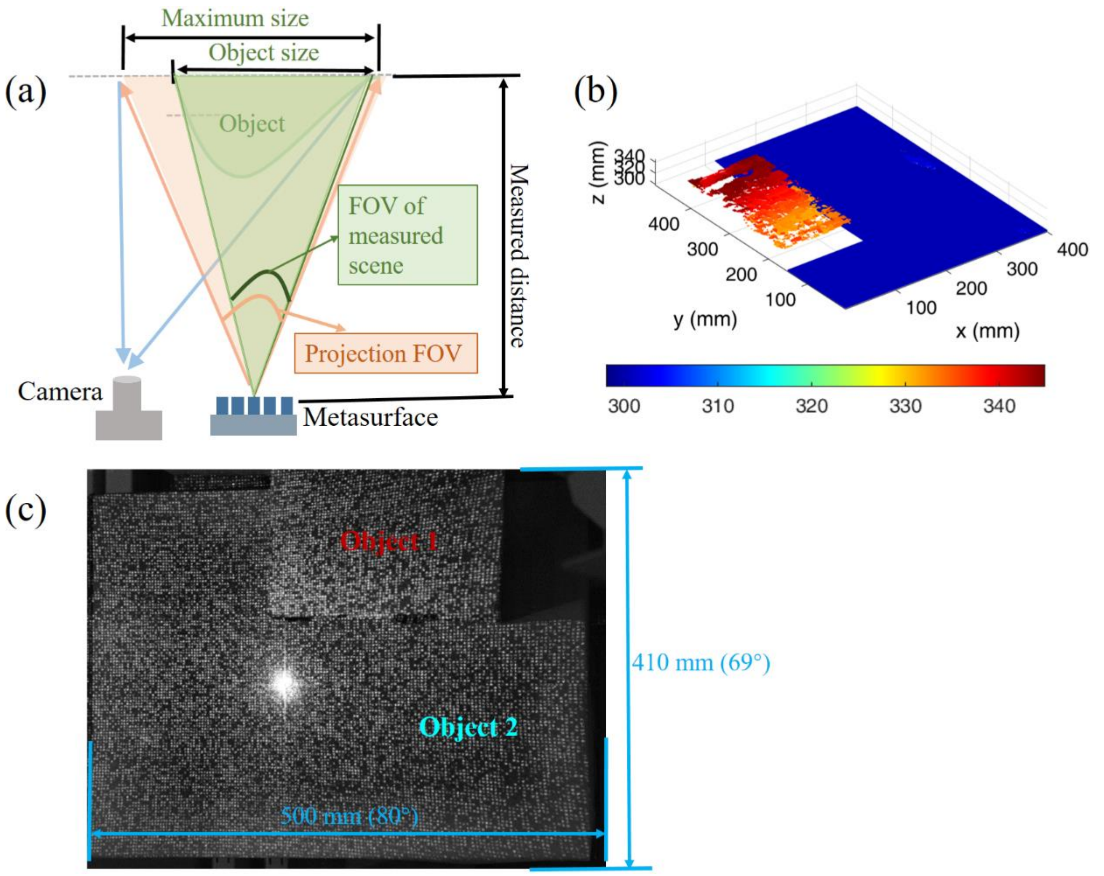

Supplementary Fig. 22 The 3D reconstruction with a larger measurement scene. (a) The schematic of measured scene and projection. (b) Reconstructed 3D point cloud maps of two objects. (c) The captured image. The measured scene is ~300 mm away from metasurface, and the measured FOV is ~ 80°× 69°.
